# Correction: Wang et al. TERT Promoter Revertant Mutation Inhibits Melanoma Growth through Intrinsic Apoptosis. *Biology* 2022, *11*, 141

**DOI:** 10.3390/biology11101400

**Published:** 2022-09-26

**Authors:** Yanbing Wang, Yiwu Chen, Chang Li, Zhiwei Xiao, Hongming Yuan, Yuanzhu Zhang, Daxin Pang, Xiaochun Tang, Mengjing Li, Hongsheng Ouyang

**Affiliations:** 1 Key Laboratory for Zoonoses Research, Ministry of Education, College of Animal Sciences, Jilin University, Changchun 130062, China; 2 College of Plant Sciences, Jilin University, Changchun 130062, China; 3 Chongqing Research Institute, Jilin University, Chongqing 401123, China; 4 Chongqing Jitang Biotechnology Research Institute, Chongqing 401123, China

The authors would like to make the following correction to the published paper [1].

## 1. Error in Main Text

We are very sorry that we misspelled a term. All the “Blc-2” in the main text should be “Bcl-2” (which means “B-cell lymphoma 2”).

## 2. Error in Figures

### 2.1. Figure 2F

In the original publication, there was a mistake in Figure 2F as published. We apologize for using the wrong image here due to an oversight. The corrected Figure 2F appears below. 
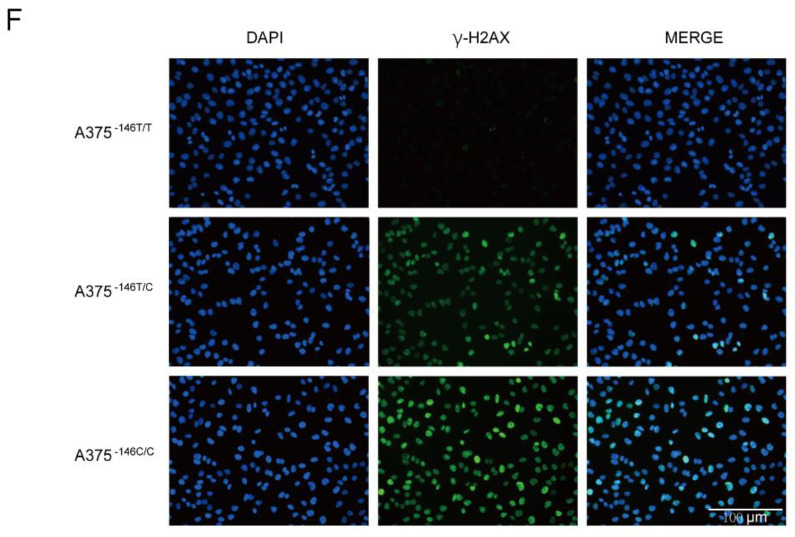


### 2.2. Figure 6O

In the original publication, there was a mistake in Figure 6O as published. VDAC1 is located in the outer mitochondrial membrane and ANT is located in the inner mitochondrial membrane. We believe that our previous image was not rigorous enough, so we have re-edited it. The corrected Figure 6O appears below. 
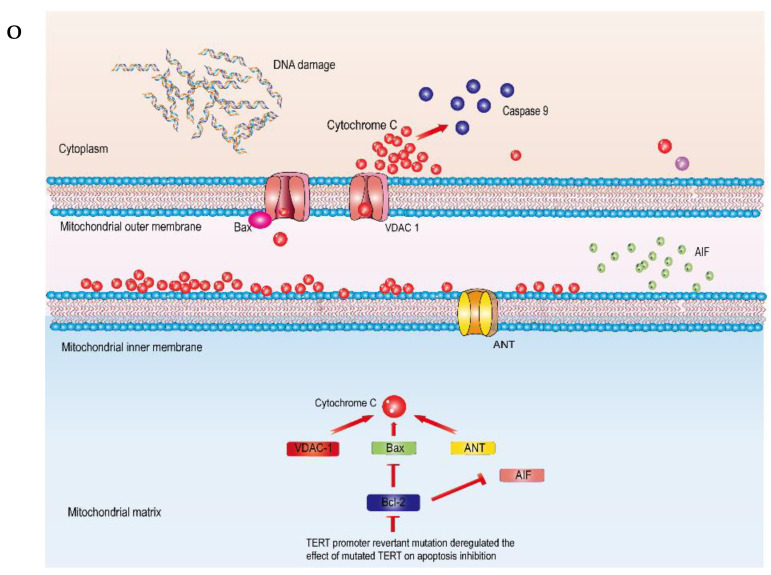


The authors apologize for any inconvenience caused and state that the scientific conclusions are unaffected. This correction was approved by the Academic Editor. The original publication has also been updated.

## References

[1] Wang Y., Chen Y., Li C., Xiao Z., Yuan H., Zhang Y., Pang D., Tang X., Li M., Ouyang H. (2022). TERT Promoter Revertant Mutation Inhibits Melanoma Growth through Intrinsic Apoptosis. Biology.

