# Supplementary material for: TERT Promoter Revertant Mutation Inhibits Melanoma Growth through Intrinsic Apoptosis
Source: Biology (Basel). 2022 Jan 14;11(1):141. doi: 10.3390/biology11010141 (PMC8773187; doi:10.3390/biology11010141)
Supplement: Supplementary file 1 [file biology-11-00141-s001.zip › biology-1512795-supplementary/Supplementary File/biology-1512795 Figure S1 and S2.pdf]

S1

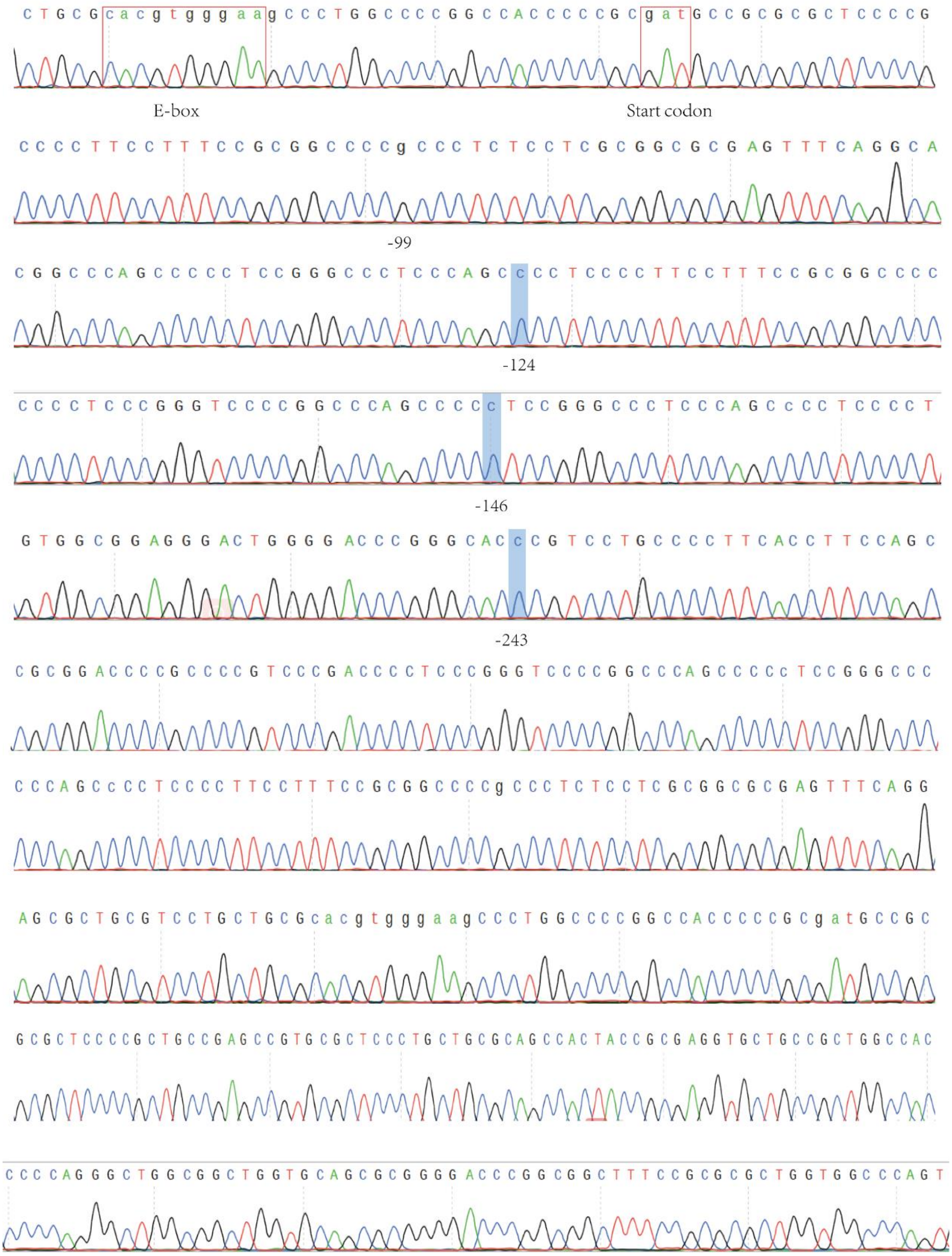


**Figure S1.** Results of sanger sequencing of the TERT promoter of A375-146C/C. Sanger sequencing of A375$^{-146C/C}$. Cells were transfected with a plasmid expressing sgRNA and SpCas9-NG. The DNA covering the most of the TERT promoter was analyzed by Sanger sequencing.

A

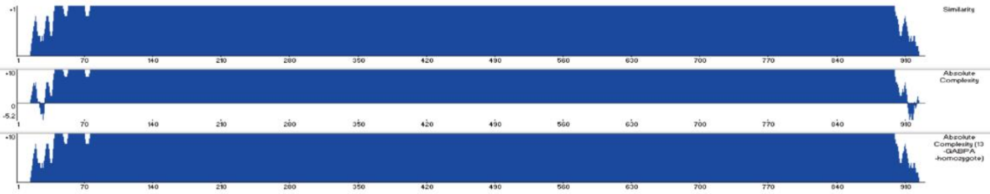


B

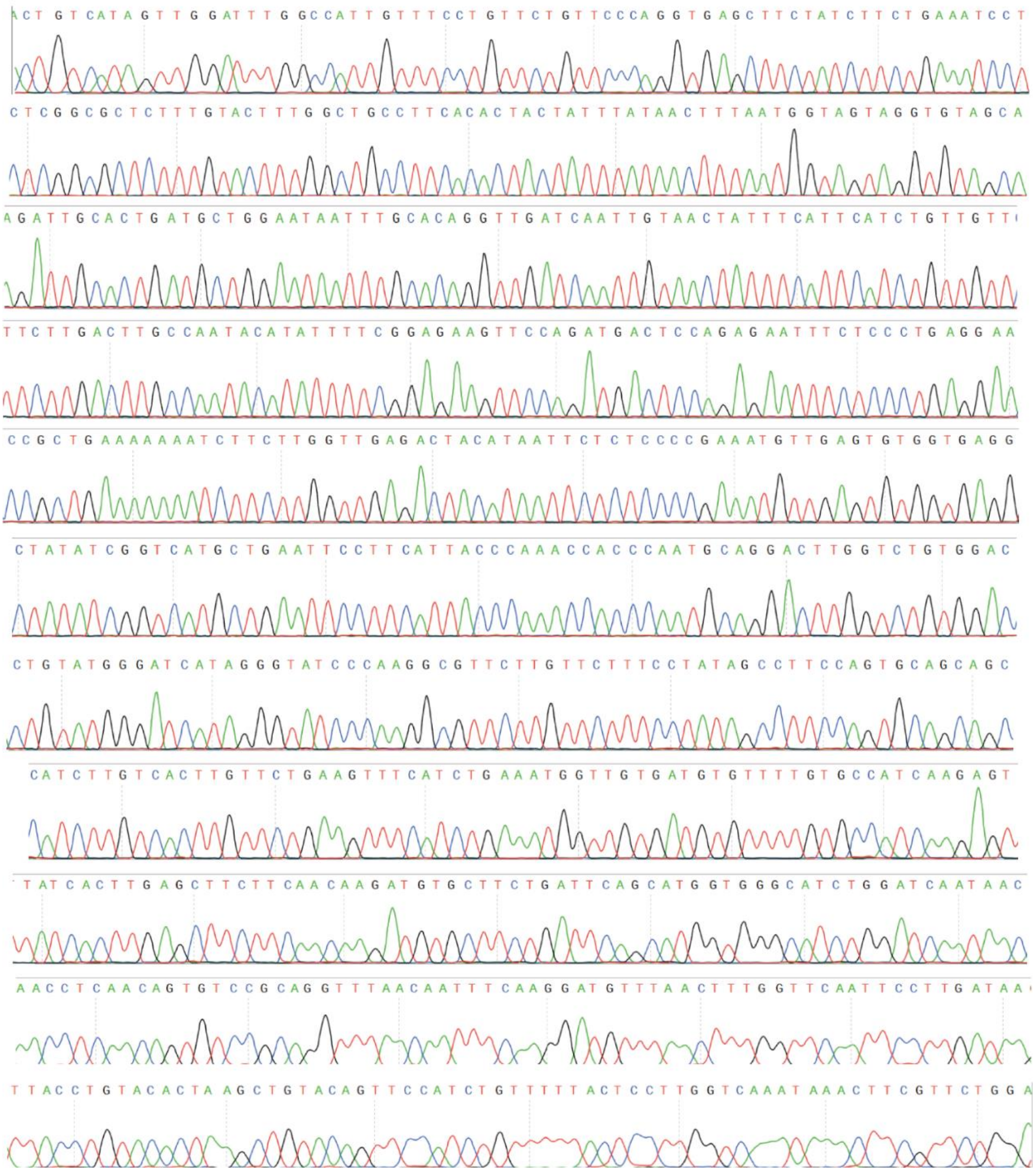

**Figure S2.** Results of sanger sequencing of GABPA of A375-146C/C. (**A**) Comparative analysis of GABPA sequencing results of A375$^{-146T/T}$ and A375$^{-146C/C}$. The DNA covering coding region in GABPA was analyzed by Sanger sequencing. (**B**) Results of sanger sequencing of the GABPA of A375$^{-146C/C}$.
