# Supplementary figures and images for: TERT Promoter Revertant Mutation Inhibits Melanoma Growth through Intrinsic Apoptosis
Source: Biology (Basel). 2022 Jan 14;11(1):141. doi: 10.3390/biology11010141 (PMC8773187; doi:10.3390/biology11010141)

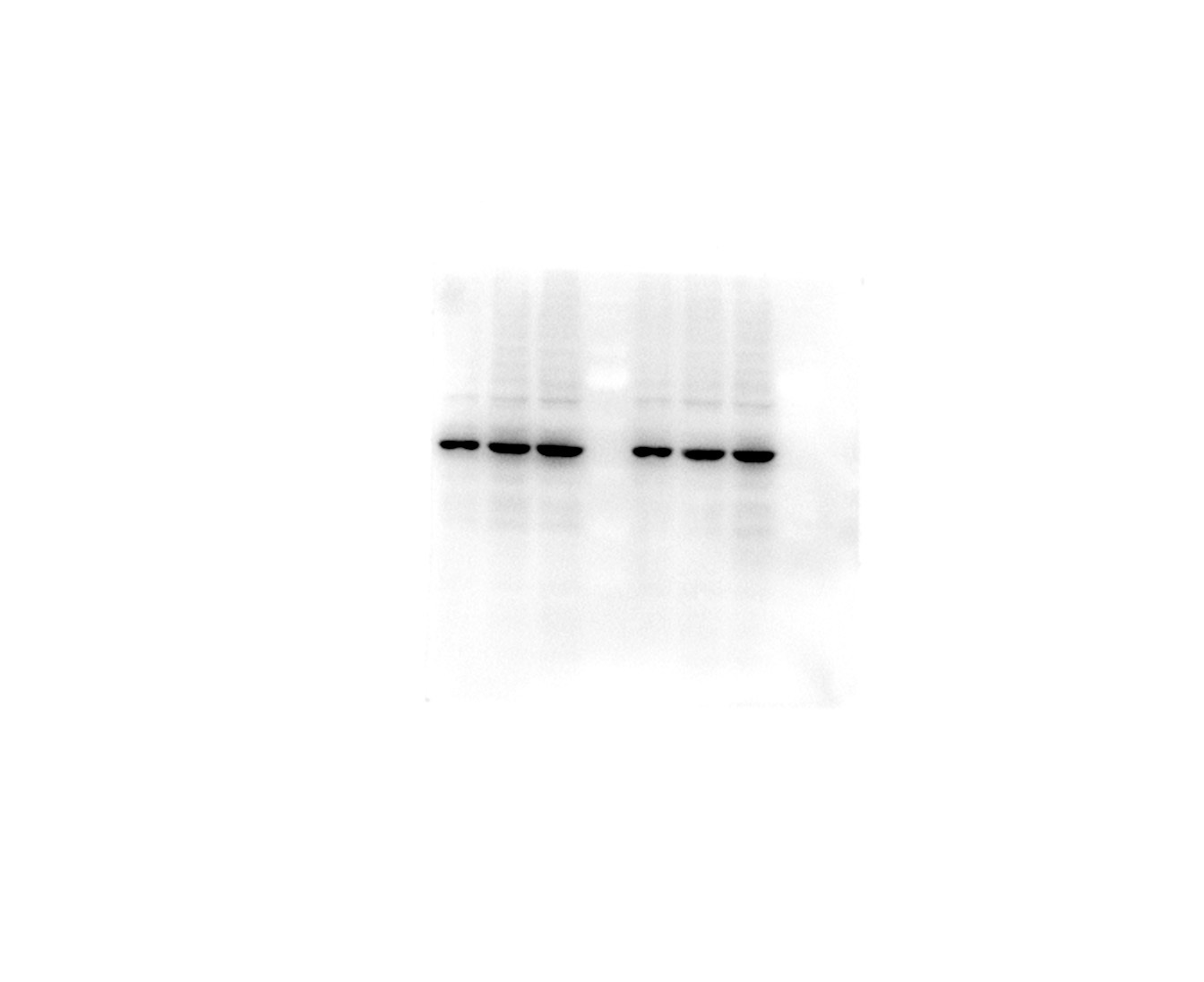

Supplement: Supplementary file 1 [file biology-11-00141-s001.zip › biology-1512795-supplementary/Supplementary File/biology-1512795 File S1/AIF/3-actin-1.jpg]

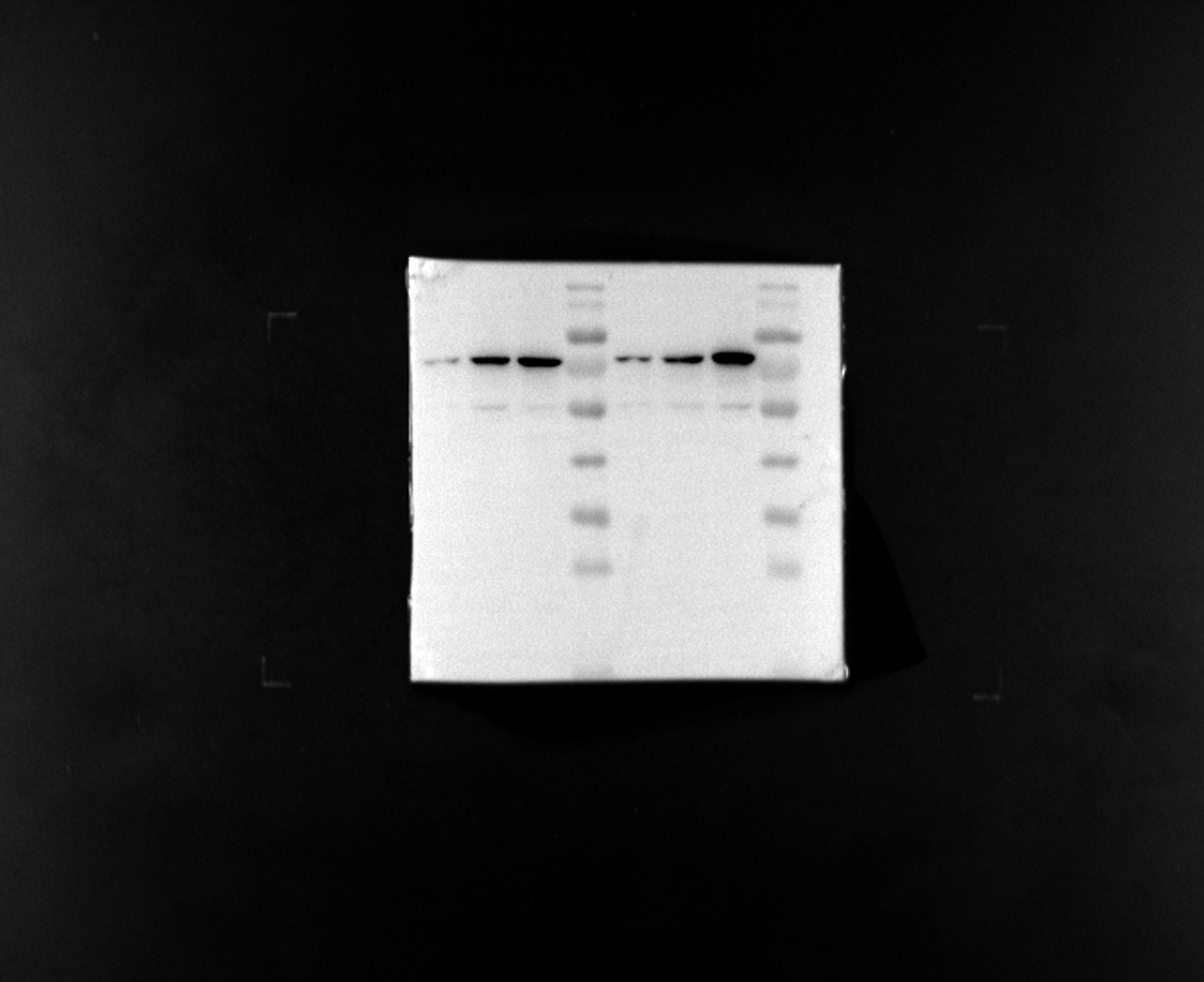

Supplement: Supplementary file 1 [file biology-11-00141-s001.zip › biology-1512795-supplementary/Supplementary File/biology-1512795 File S1/AIF/3-AIF.Tif]

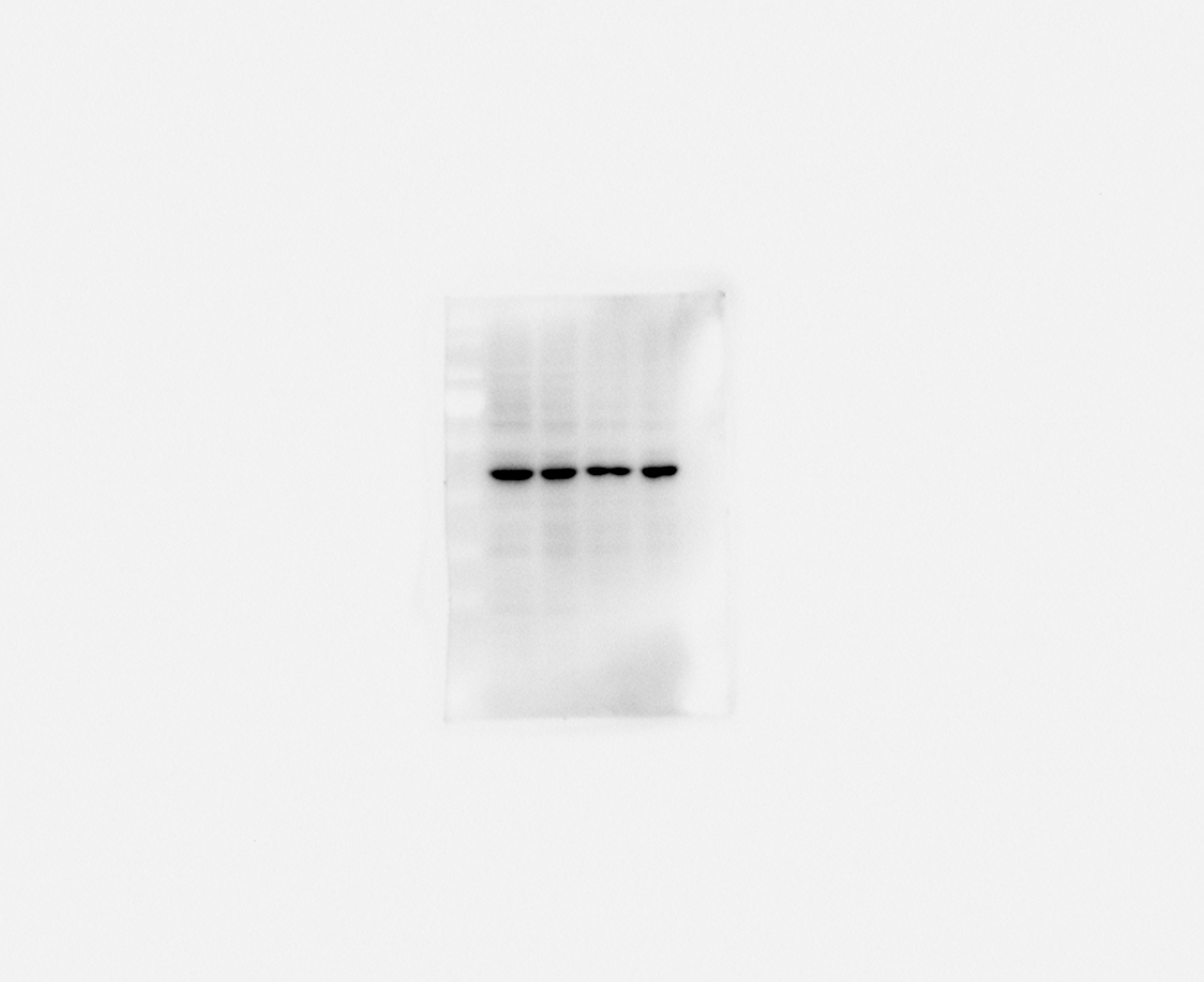

Supplement: Supplementary file 1 [file biology-11-00141-s001.zip › biology-1512795-supplementary/Supplementary File/biology-1512795 File S1/ANT/2-actin-1.Tif]

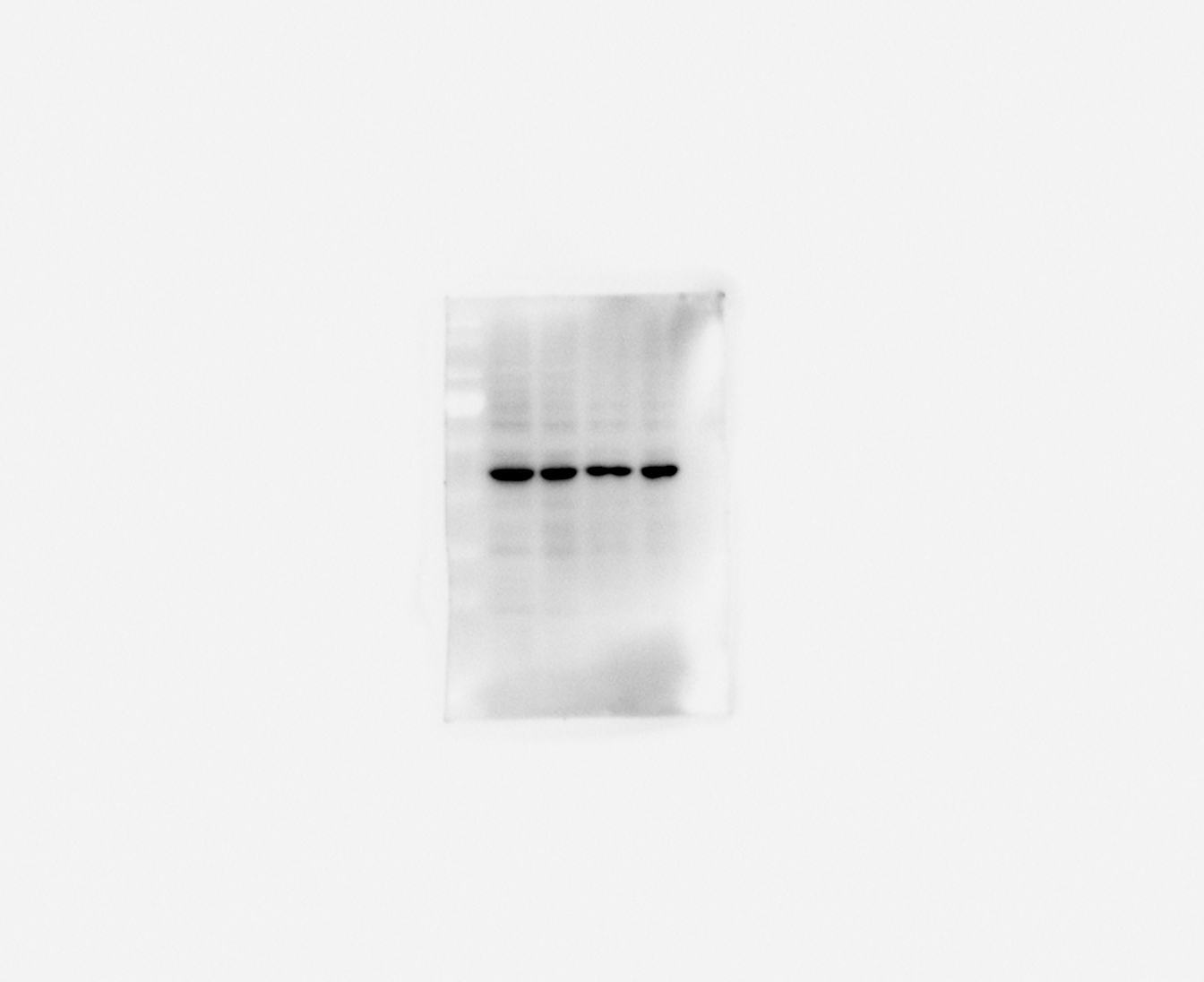

Supplement: Supplementary file 1 [file biology-11-00141-s001.zip › biology-1512795-supplementary/Supplementary File/biology-1512795 File S1/ANT/2-actin.Tif]

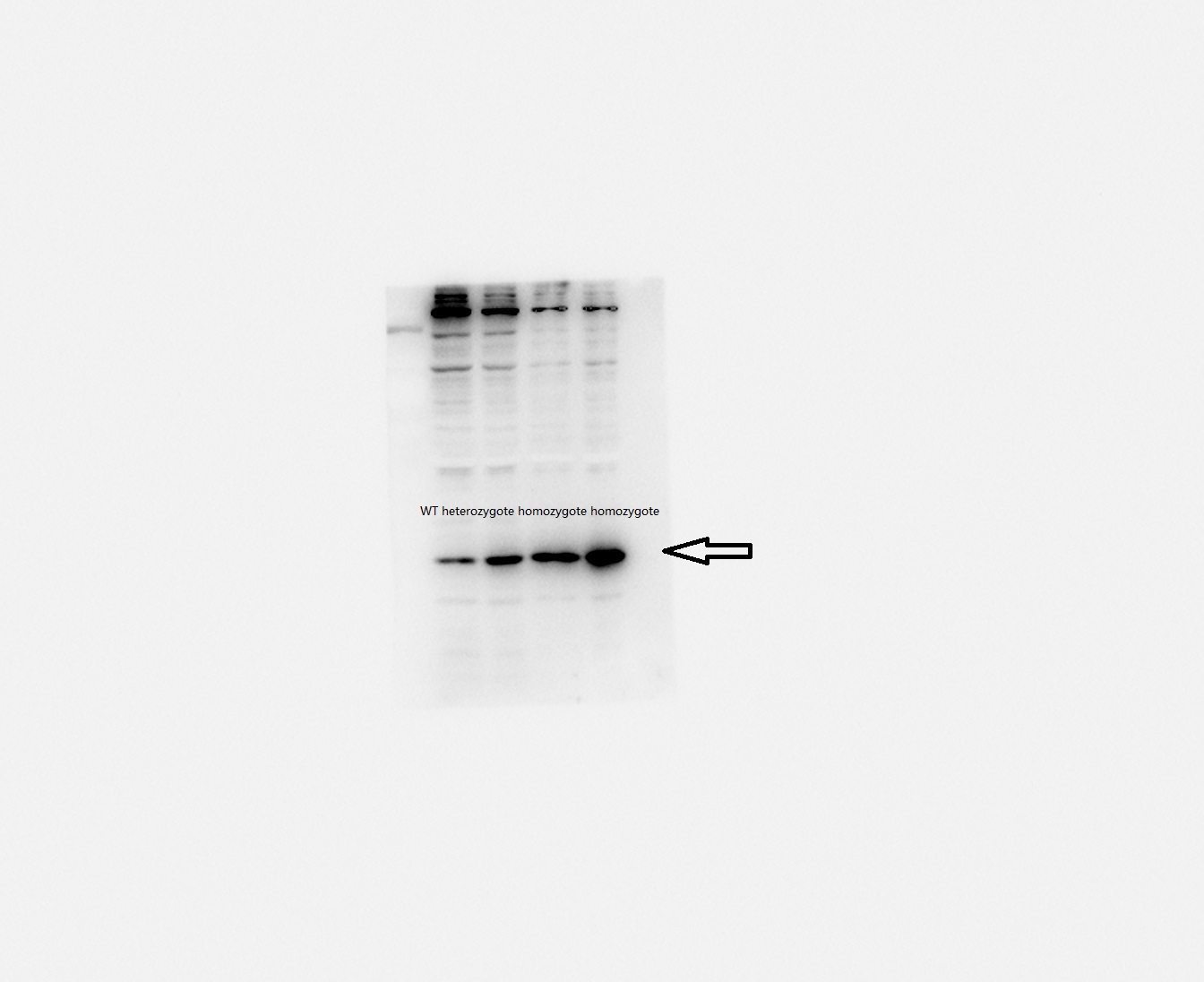

Supplement: Supplementary file 1 [file biology-11-00141-s001.zip › biology-1512795-supplementary/Supplementary File/biology-1512795 File S1/ANT/2-ANT-1.jpg]

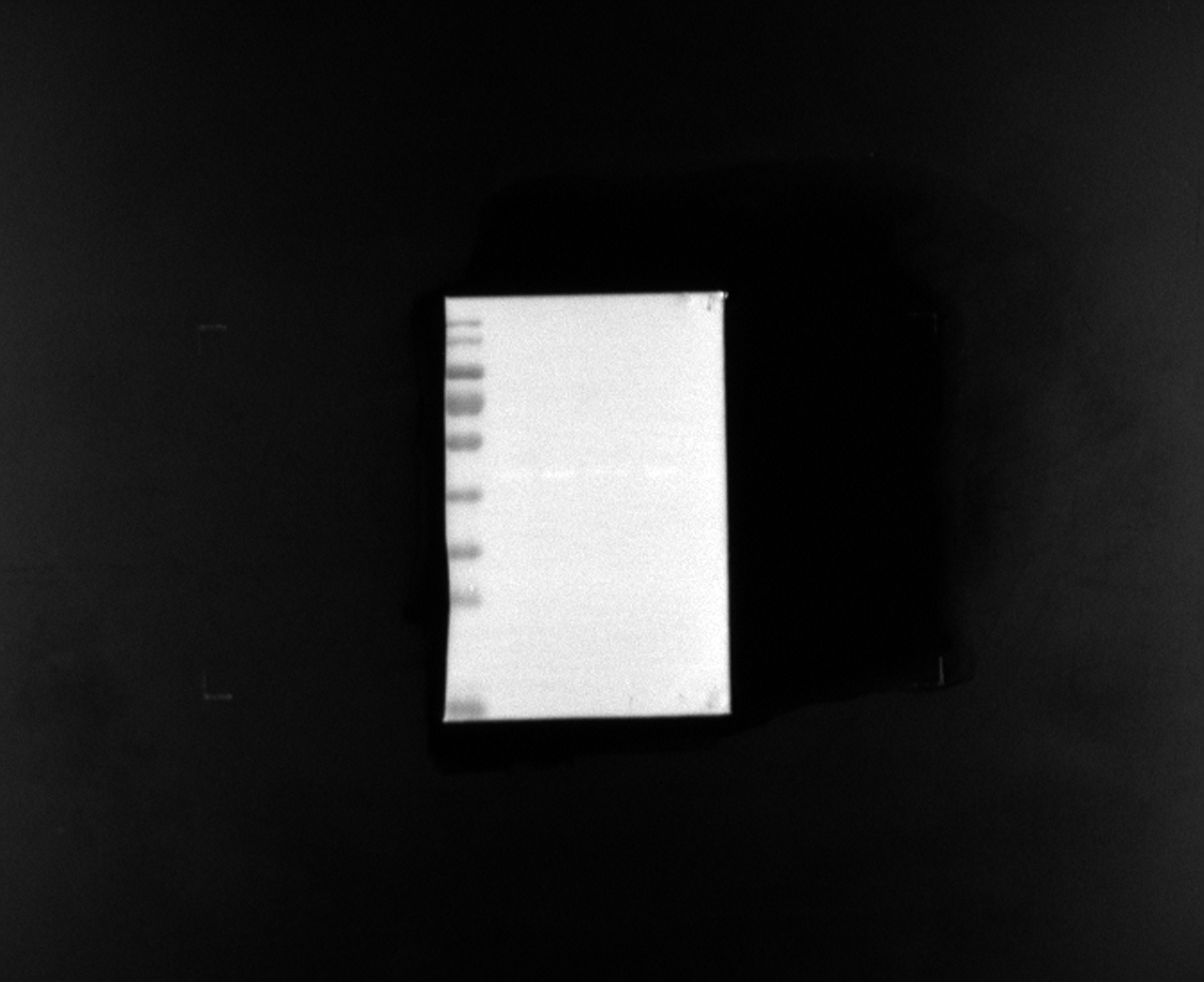

Supplement: Supplementary file 1 [file biology-11-00141-s001.zip › biology-1512795-supplementary/Supplementary File/biology-1512795 File S1/ANT/2-marker.jpg]

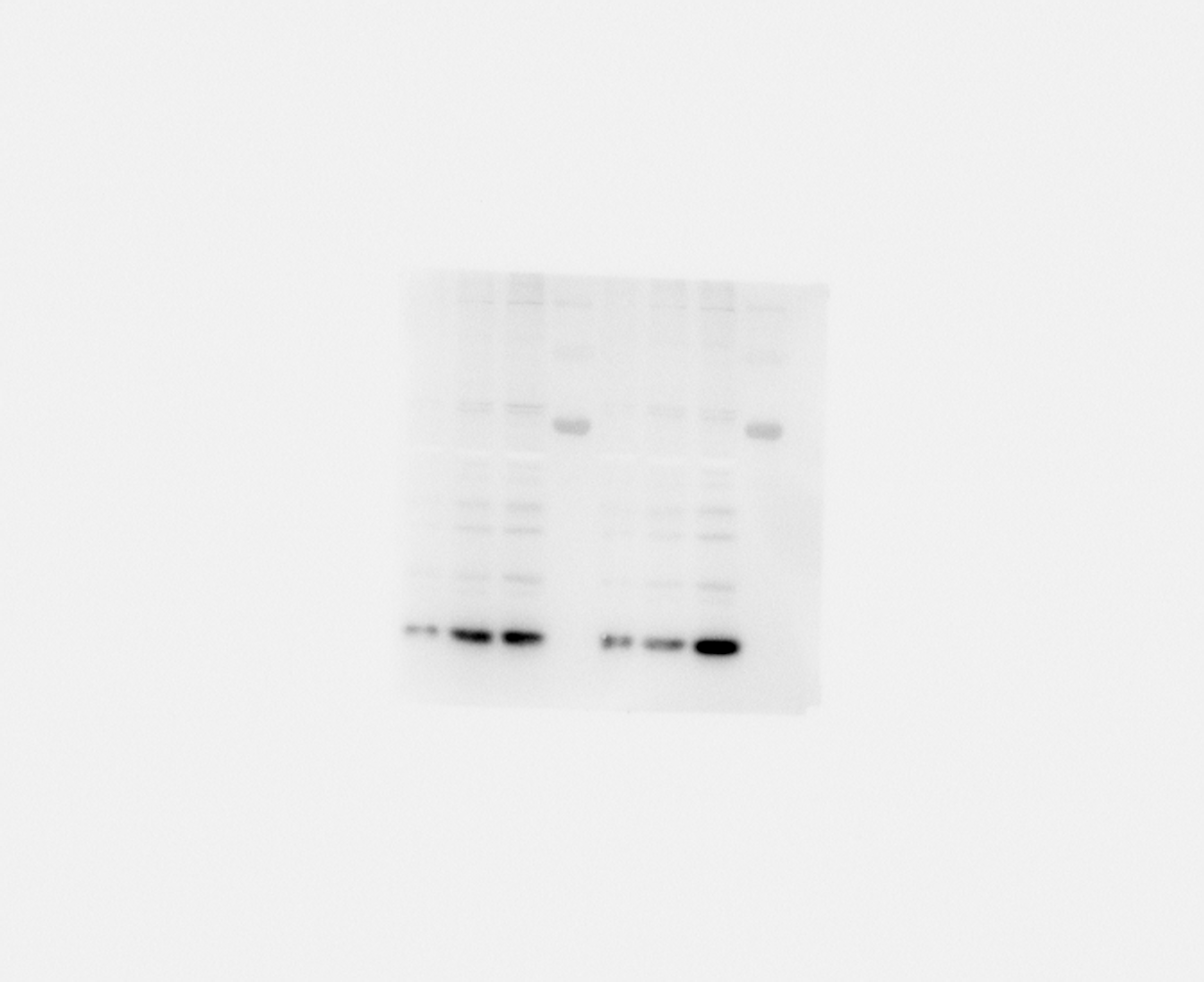

Supplement: Supplementary file 1 [file biology-11-00141-s001.zip › biology-1512795-supplementary/Supplementary File/biology-1512795 File S1/Bax/3-Bax-2.Tif]

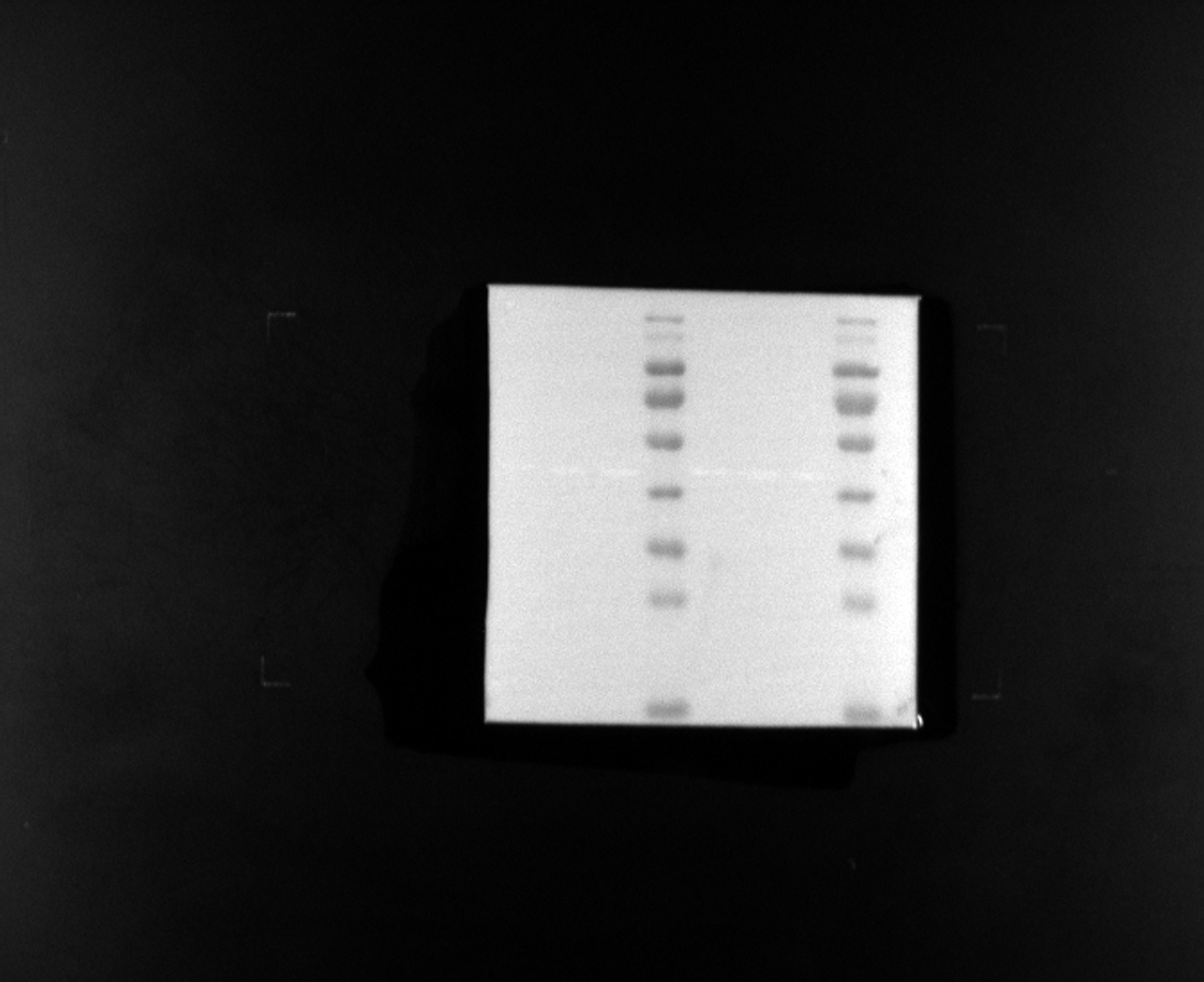

Supplement: Supplementary file 1 [file biology-11-00141-s001.zip › biology-1512795-supplementary/Supplementary File/biology-1512795 File S1/Bax/marker.jpg]

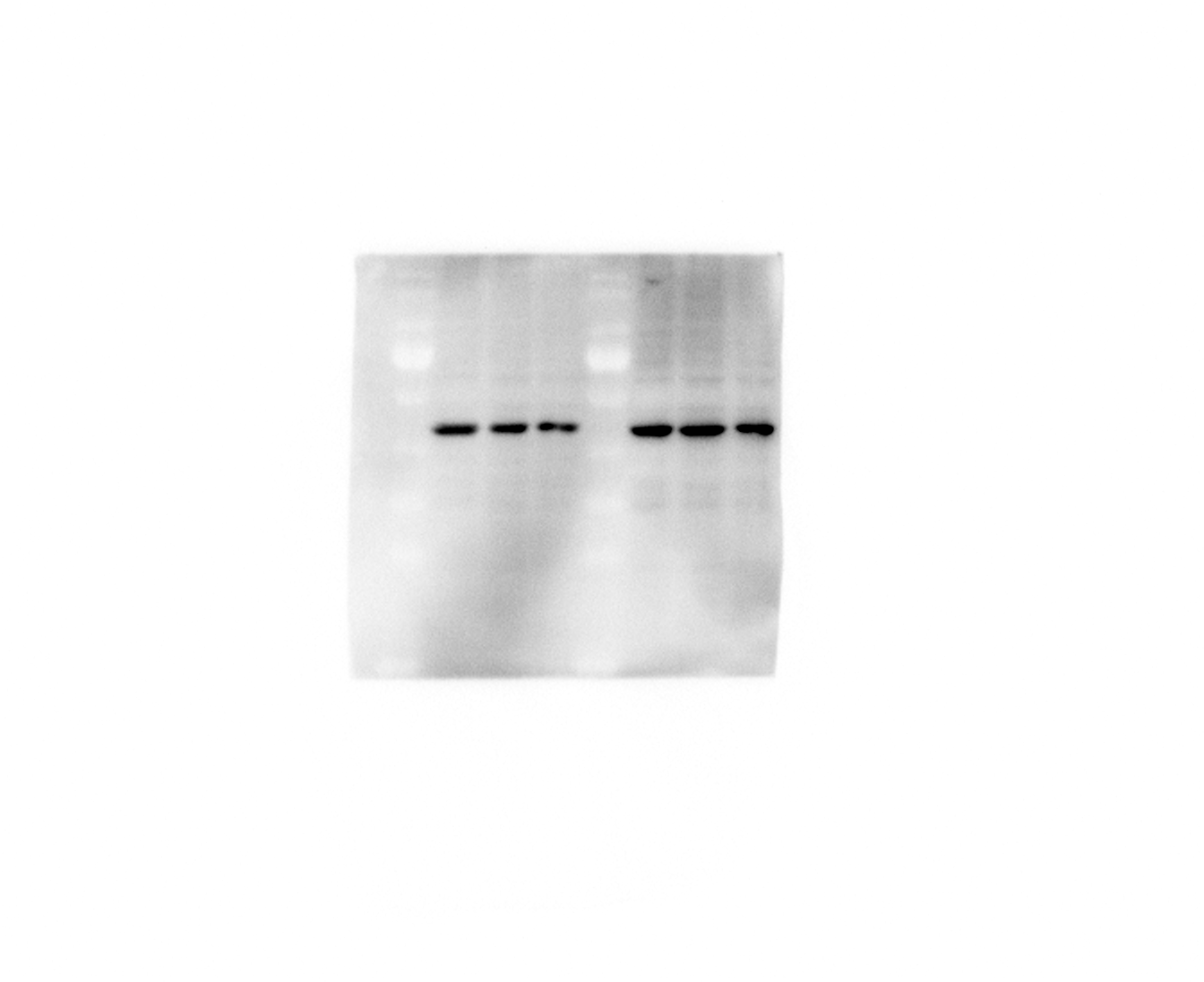

Supplement: Supplementary file 1 [file biology-11-00141-s001.zip › biology-1512795-supplementary/Supplementary File/biology-1512795 File S1/Blc-2/1-actin-2.Tif]

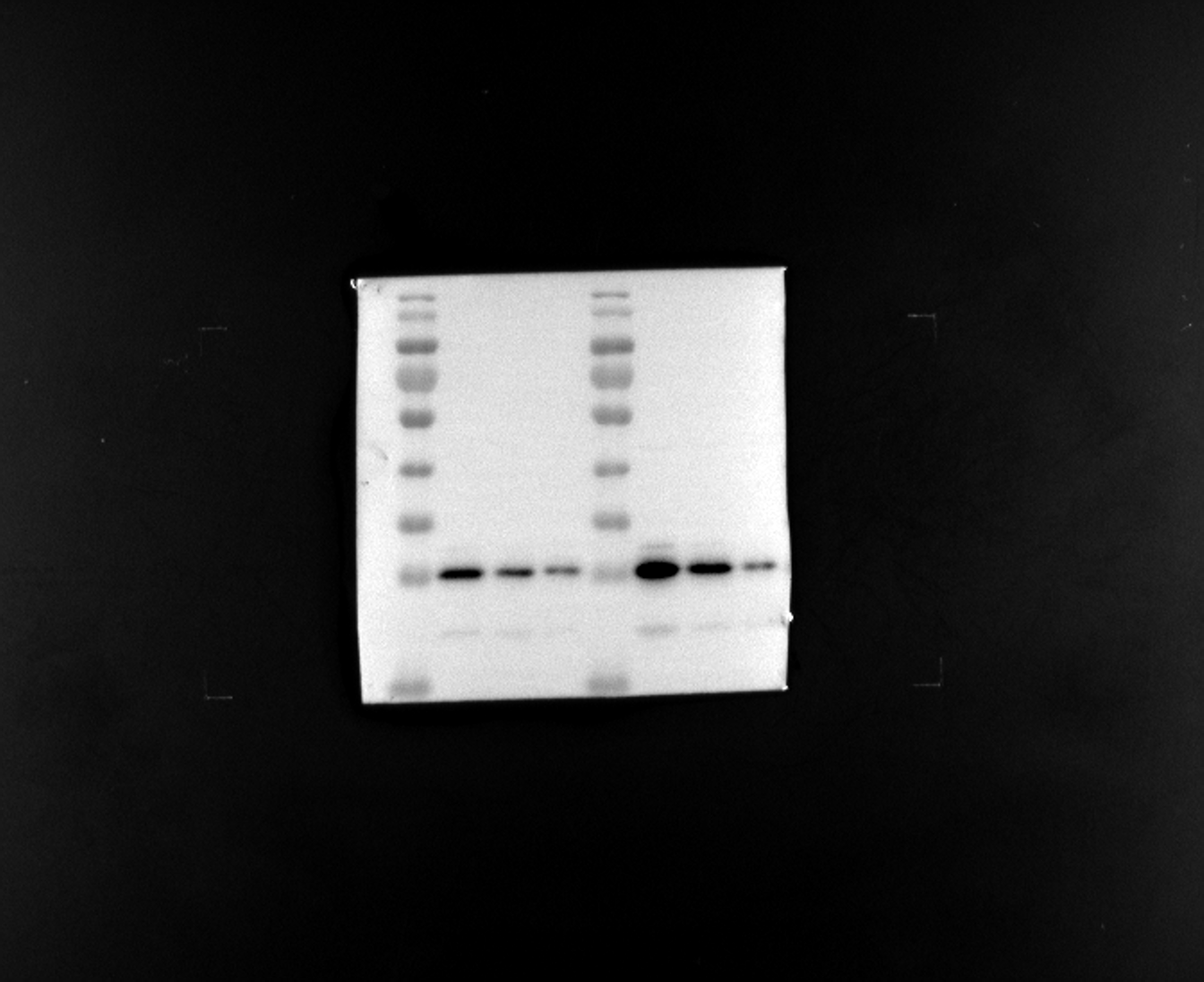

Supplement: Supplementary file 1 [file biology-11-00141-s001.zip › biology-1512795-supplementary/Supplementary File/biology-1512795 File S1/Blc-2/1-Blc-2 -3.Tif]

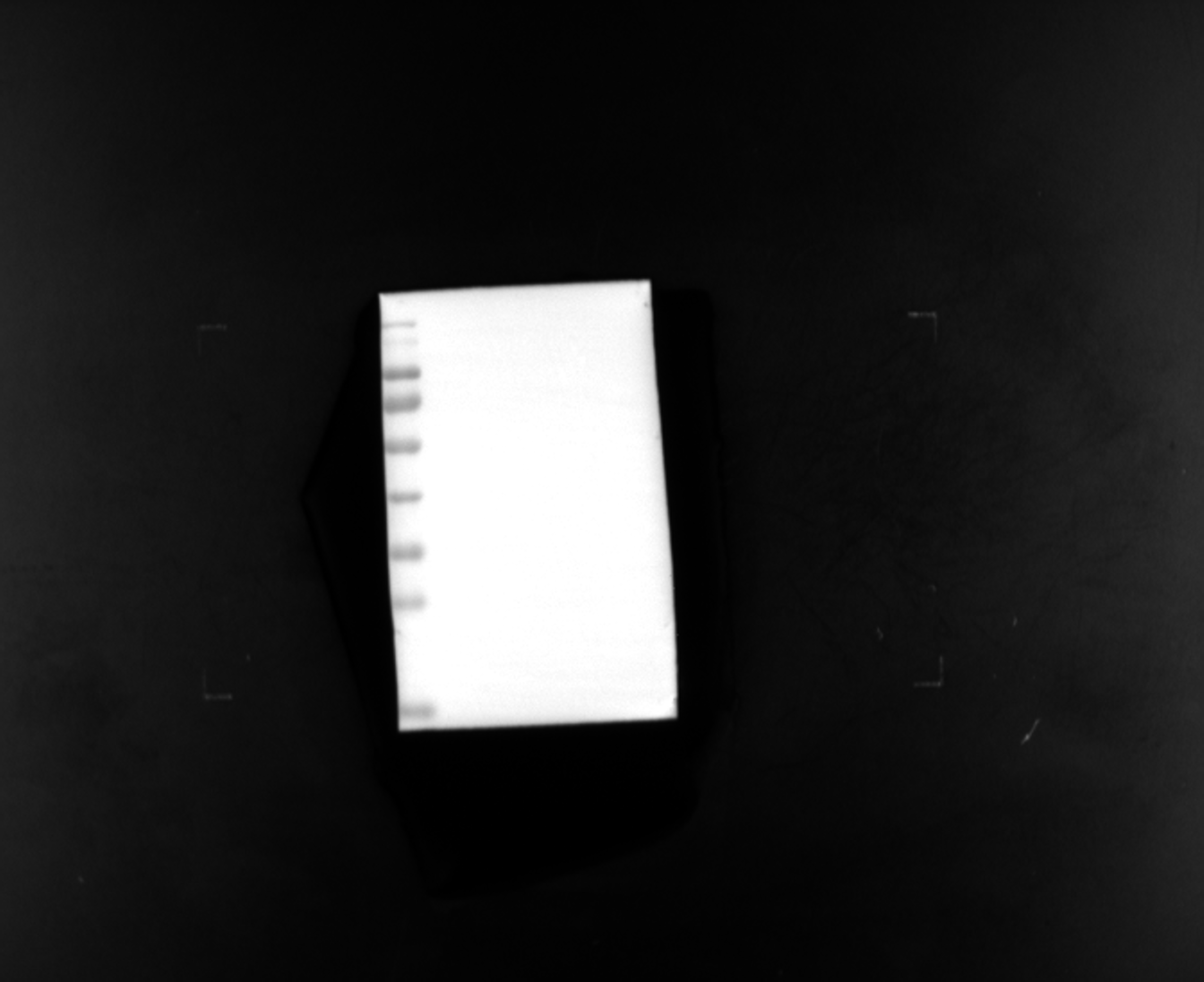

Supplement: Supplementary file 1 [file biology-11-00141-s001.zip › biology-1512795-supplementary/Supplementary File/biology-1512795 File S1/c-MYC/MYC-3 marker.Tif]

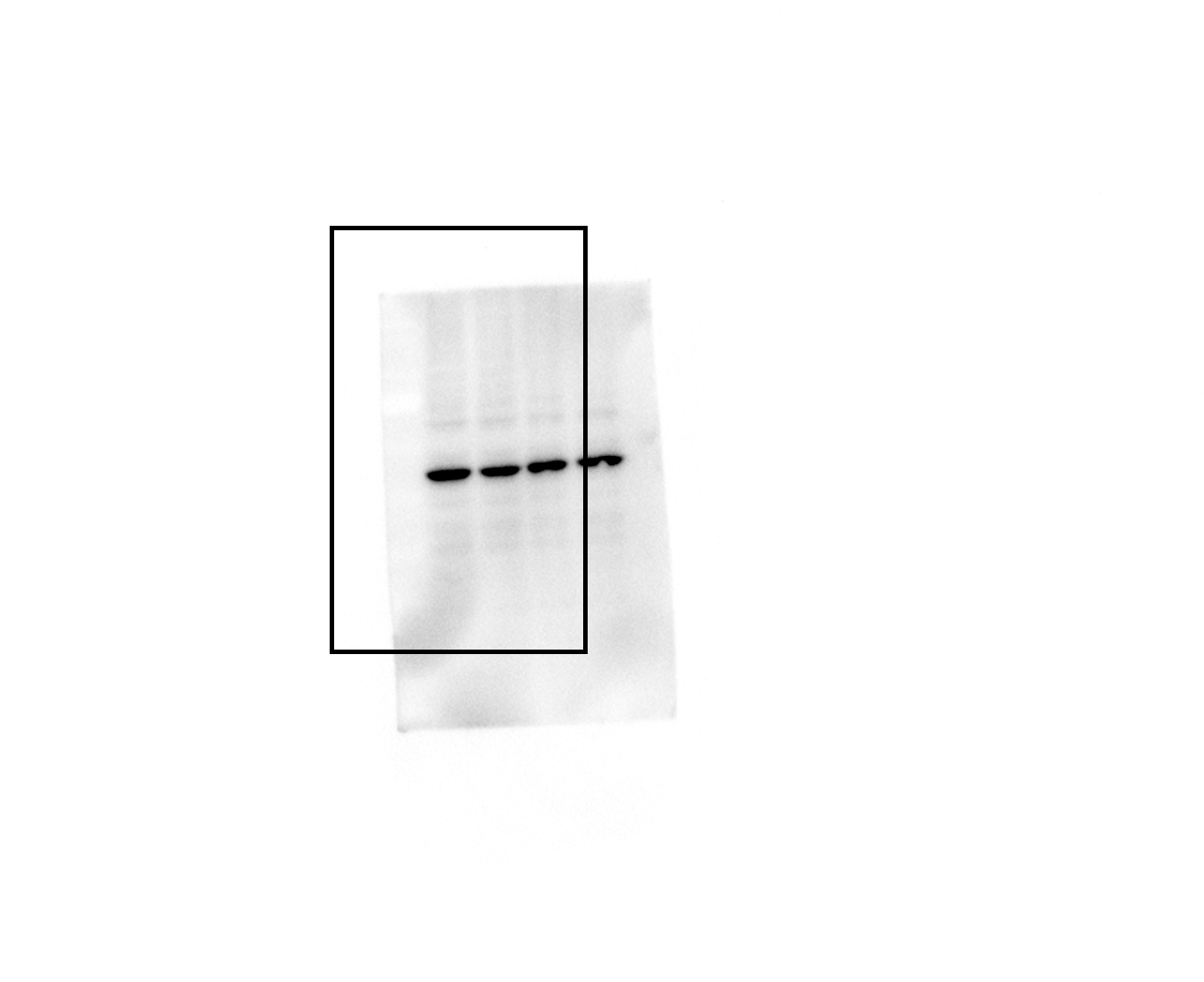

Supplement: Supplementary file 1 [file biology-11-00141-s001.zip › biology-1512795-supplementary/Supplementary File/biology-1512795 File S1/c-MYC/MYC-3-actin.Tif]

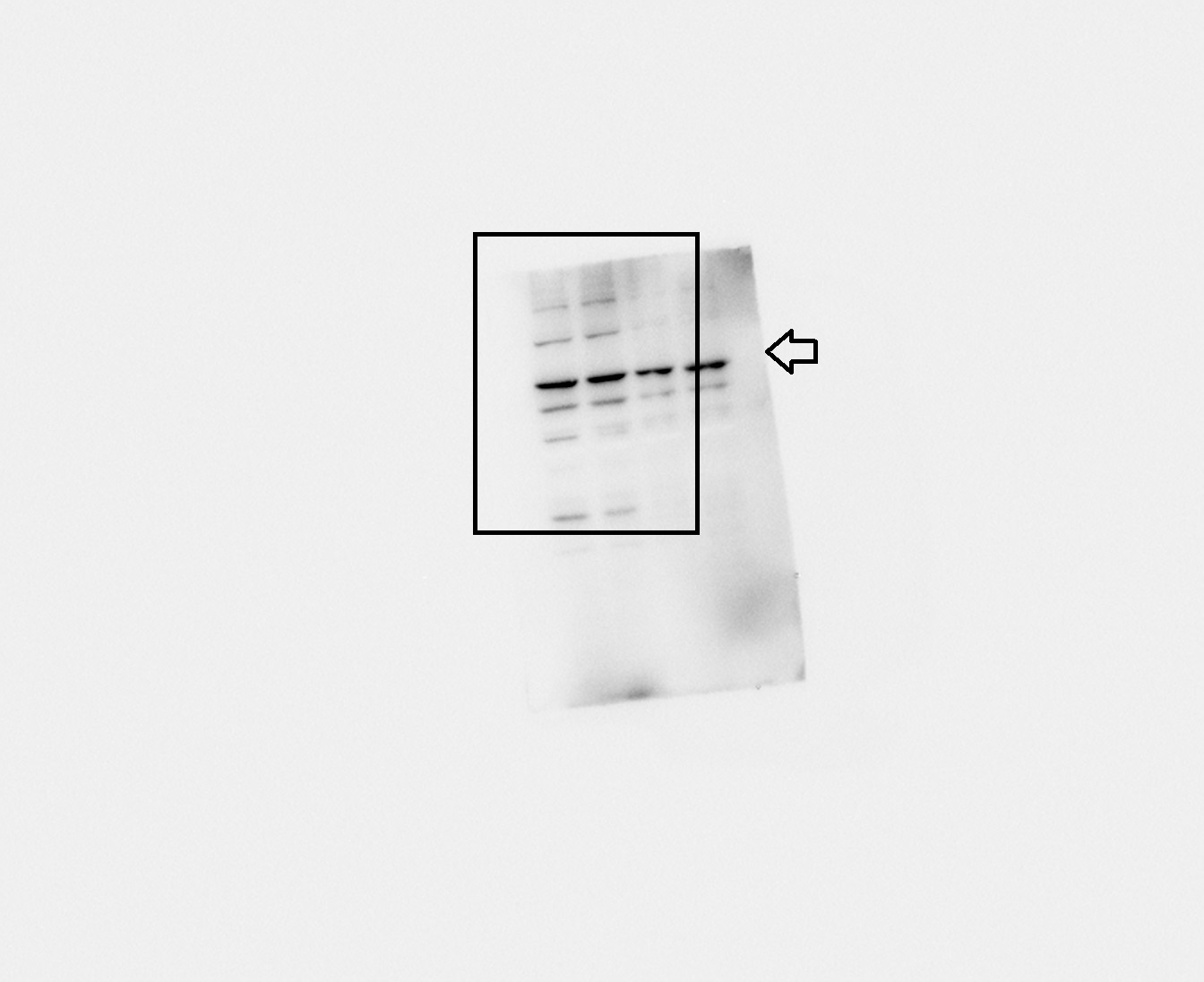

Supplement: Supplementary file 1 [file biology-11-00141-s001.zip › biology-1512795-supplementary/Supplementary File/biology-1512795 File S1/c-MYC/MYC-3.jpg]

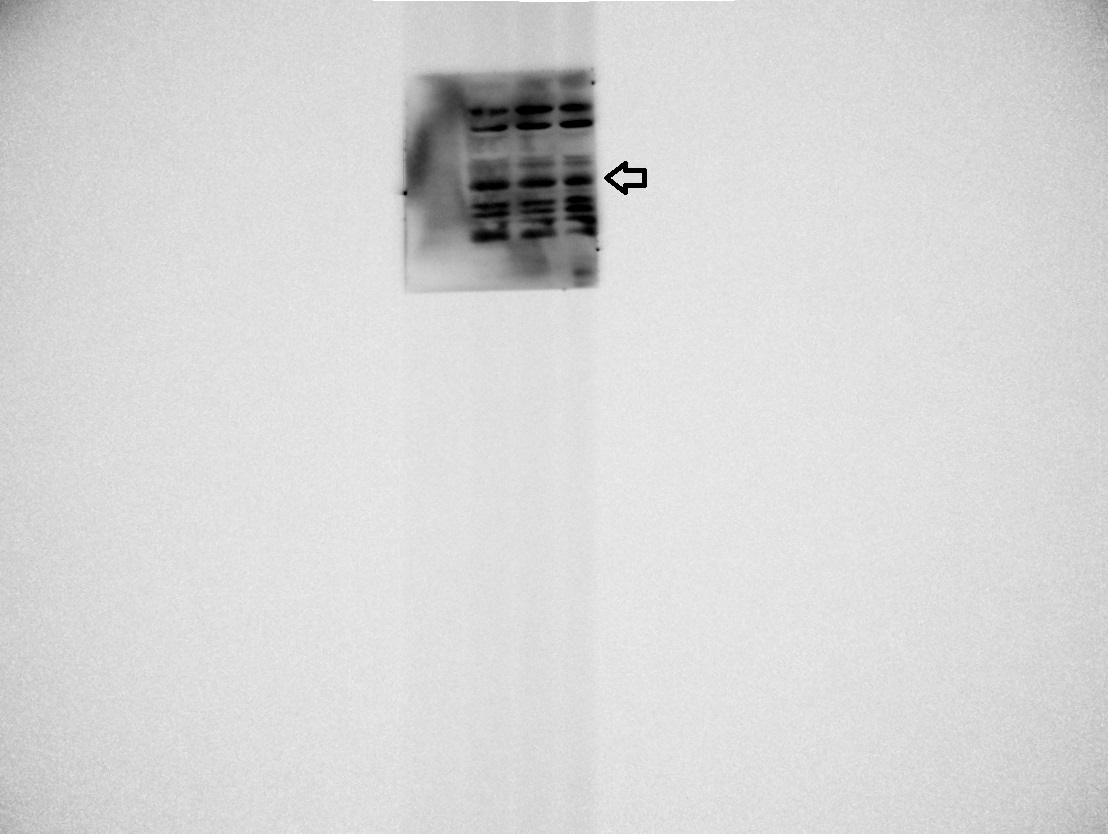

Supplement: Supplementary file 1 [file biology-11-00141-s001.zip › biology-1512795-supplementary/Supplementary File/biology-1512795 File S1/Caspase 9/Caspase 9-1 actin.jpg]

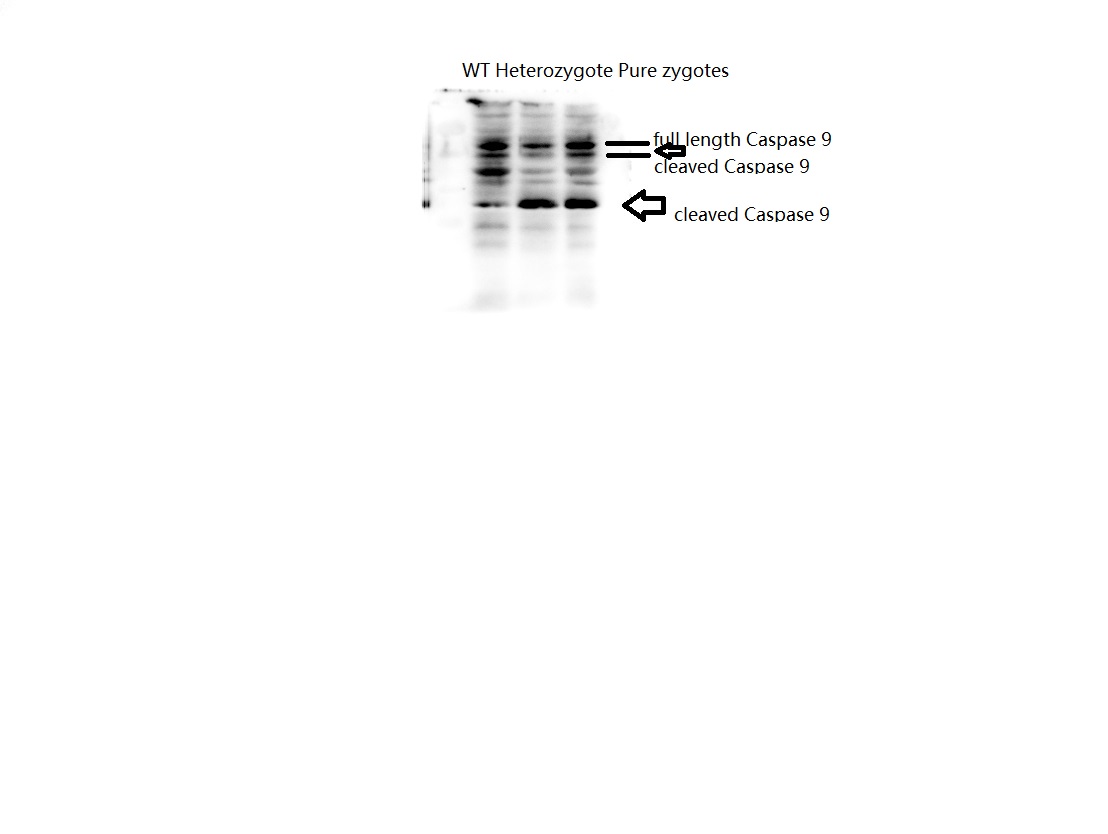

Supplement: Supplementary file 1 [file biology-11-00141-s001.zip › biology-1512795-supplementary/Supplementary File/biology-1512795 File S1/Caspase 9/Caspase 9-1.jpg]

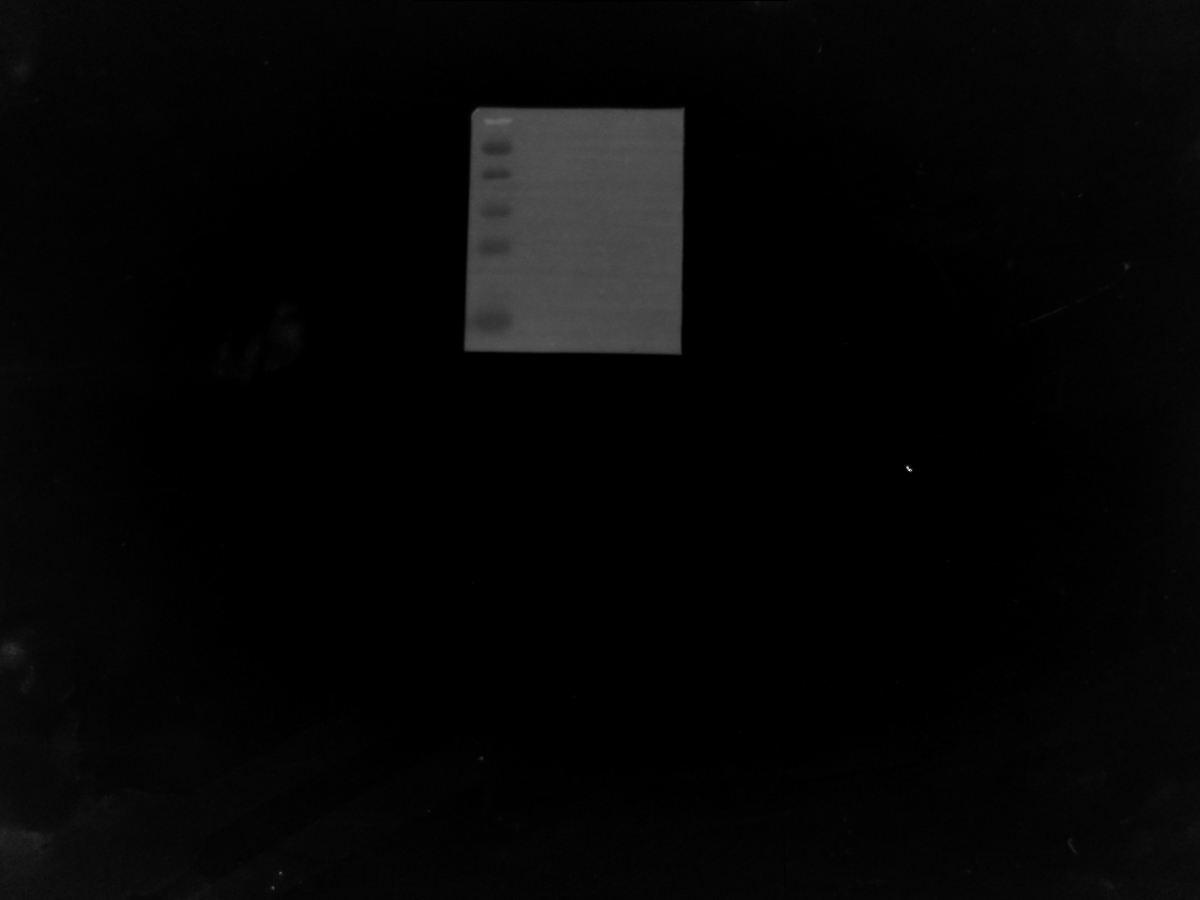

Supplement: Supplementary file 1 [file biology-11-00141-s001.zip › biology-1512795-supplementary/Supplementary File/biology-1512795 File S1/Caspase 9/Caspase9 -1 marker.jpg]

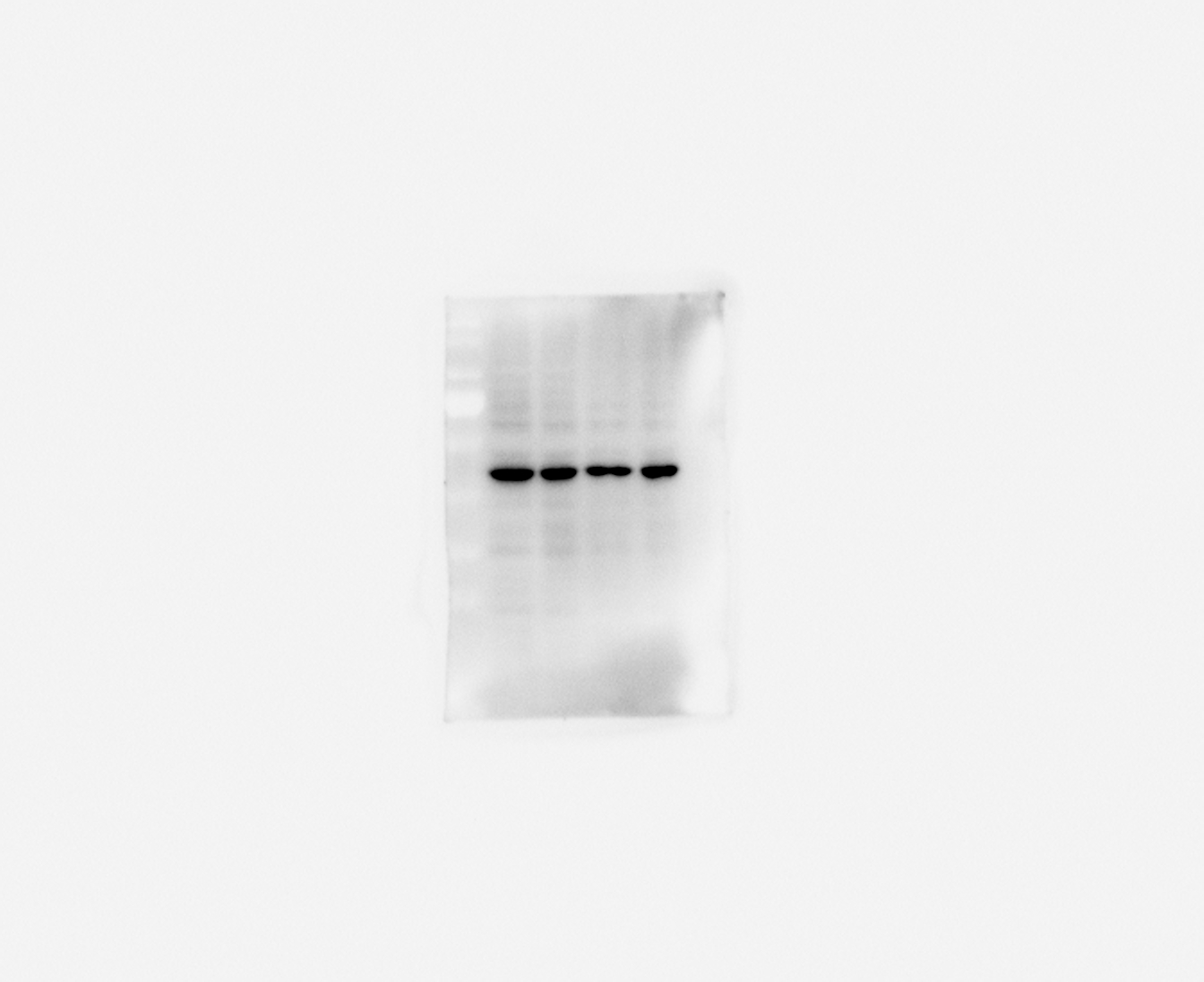

Supplement: Supplementary file 1 [file biology-11-00141-s001.zip › biology-1512795-supplementary/Supplementary File/biology-1512795 File S1/catenin/catenin-3-actin.jpg]

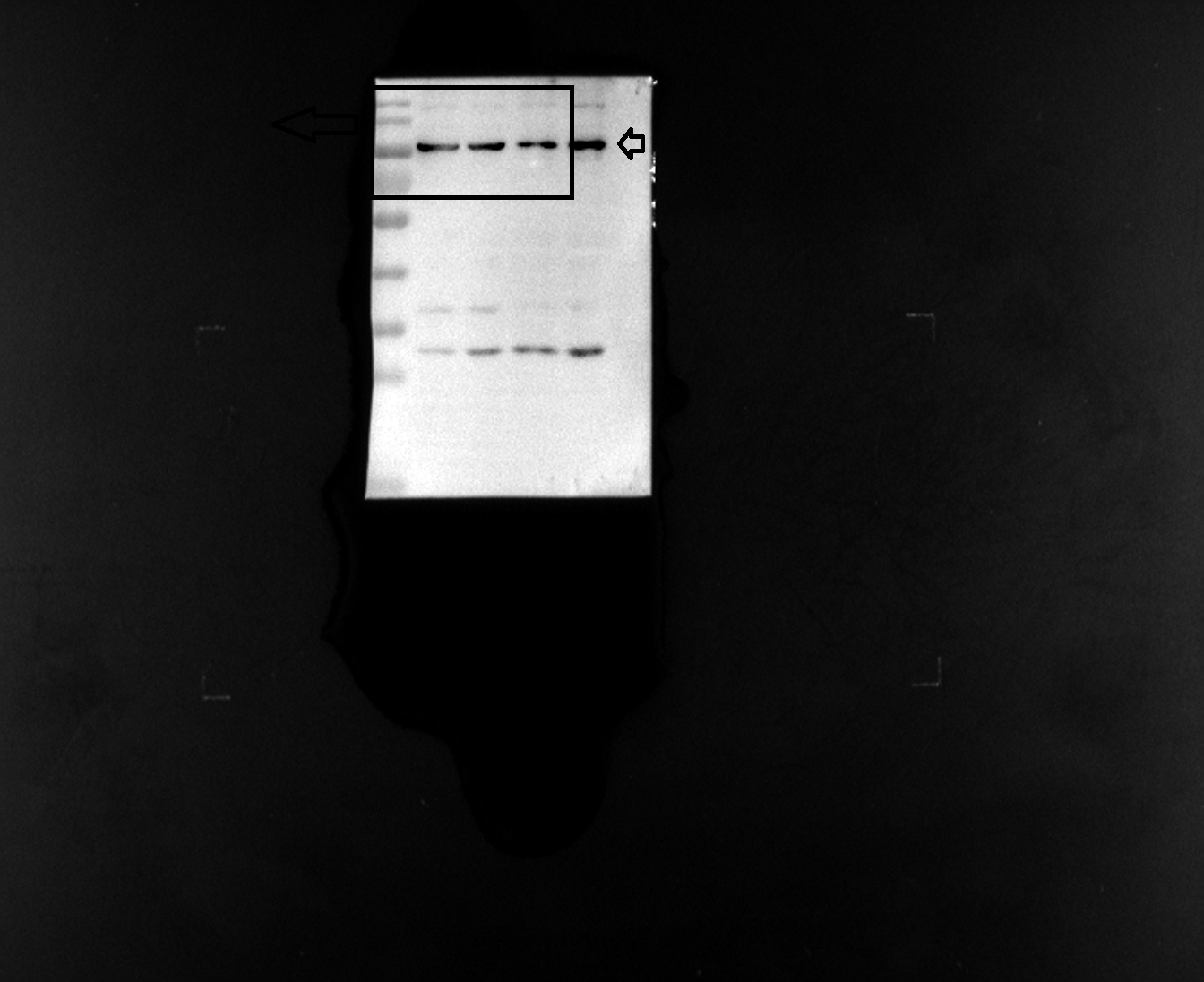

Supplement: Supplementary file 1 [file biology-11-00141-s001.zip › biology-1512795-supplementary/Supplementary File/biology-1512795 File S1/catenin/catenin.Tif]

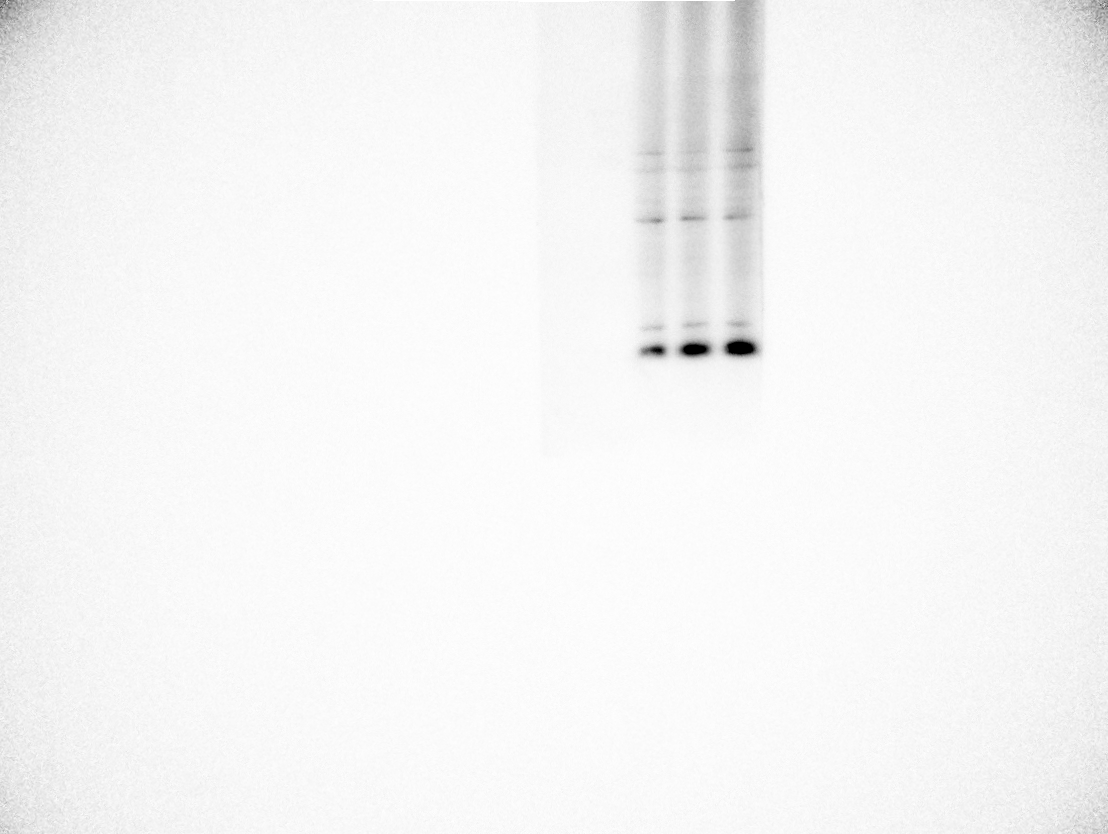

Supplement: Supplementary file 1 [file biology-11-00141-s001.zip › biology-1512795-supplementary/Supplementary File/biology-1512795 File S1/Cyt c/6 Cyt c.jpg]

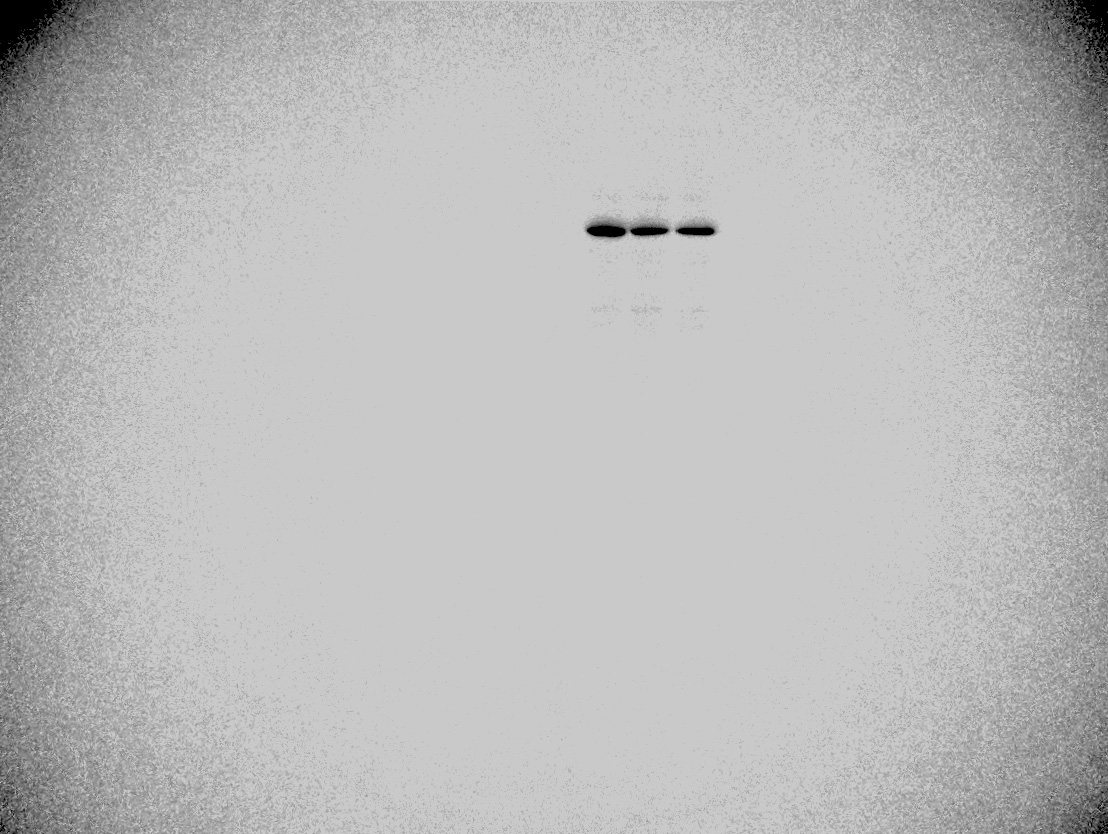

Supplement: Supplementary file 1 [file biology-11-00141-s001.zip › biology-1512795-supplementary/Supplementary File/biology-1512795 File S1/Cyt c/6-Cytc actin.jpg]

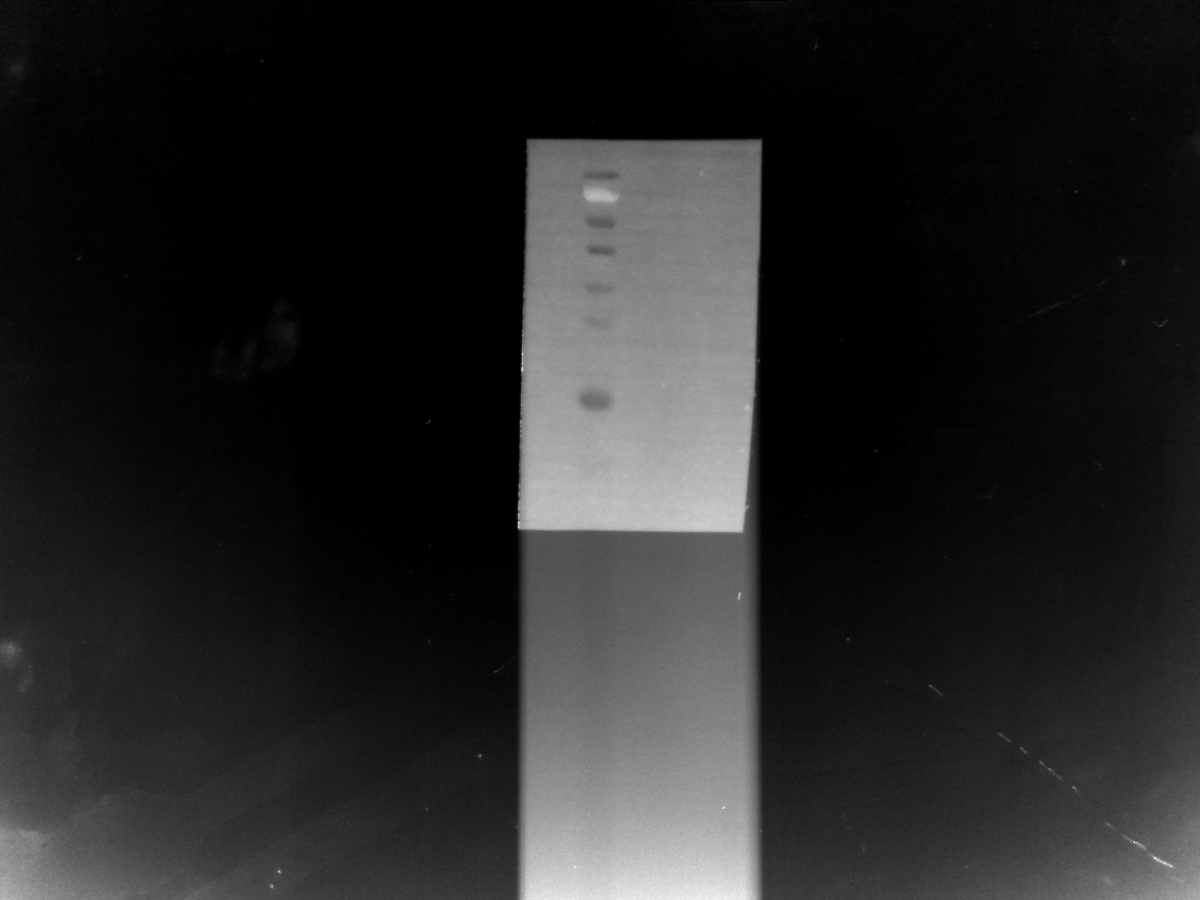

Supplement: Supplementary file 1 [file biology-11-00141-s001.zip › biology-1512795-supplementary/Supplementary File/biology-1512795 File S1/Cyt c/6marker.jpg]

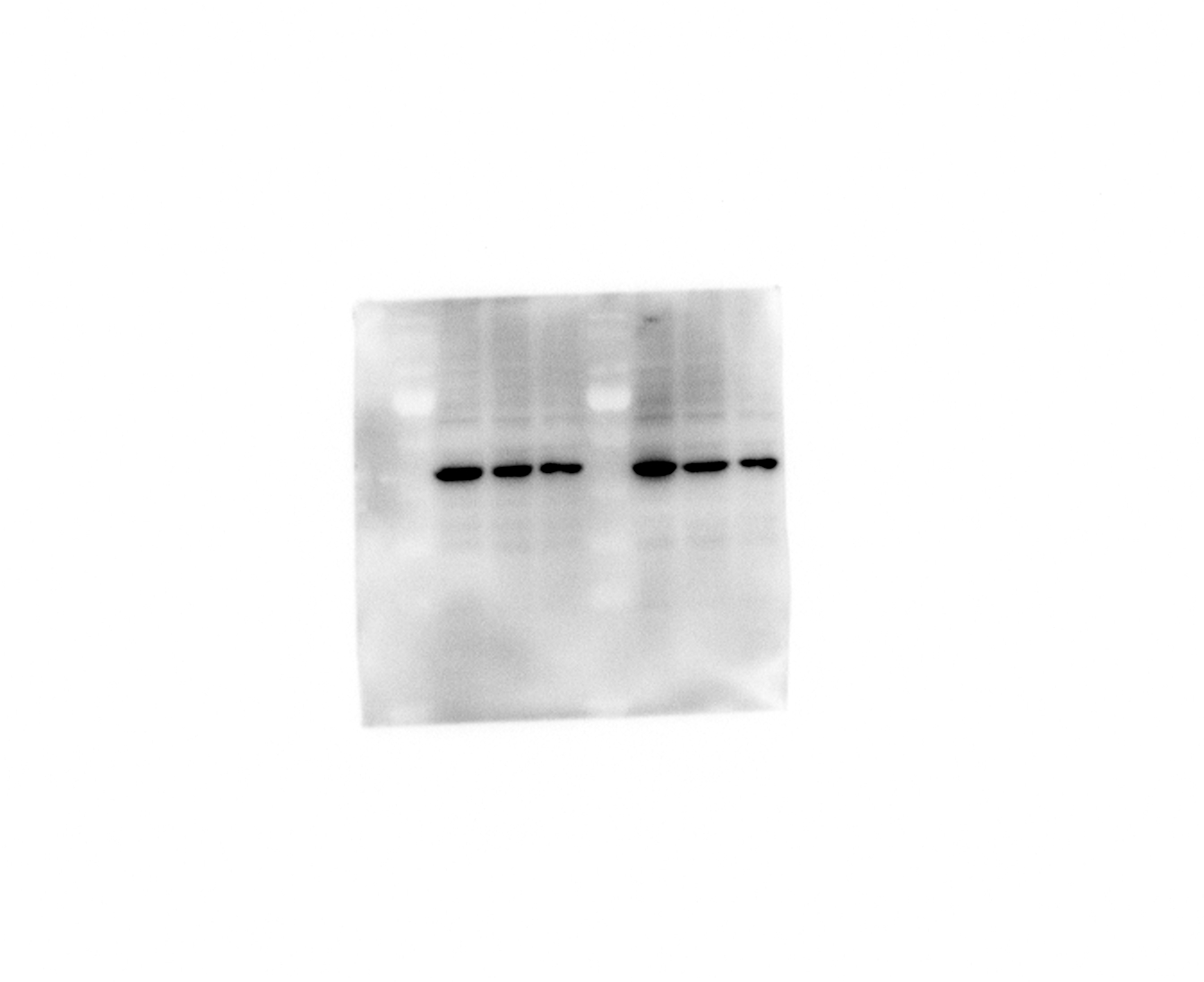

Supplement: Supplementary file 1 [file biology-11-00141-s001.zip › biology-1512795-supplementary/Supplementary File/biology-1512795 File S1/ETS/ETS-1 actin.jpg]

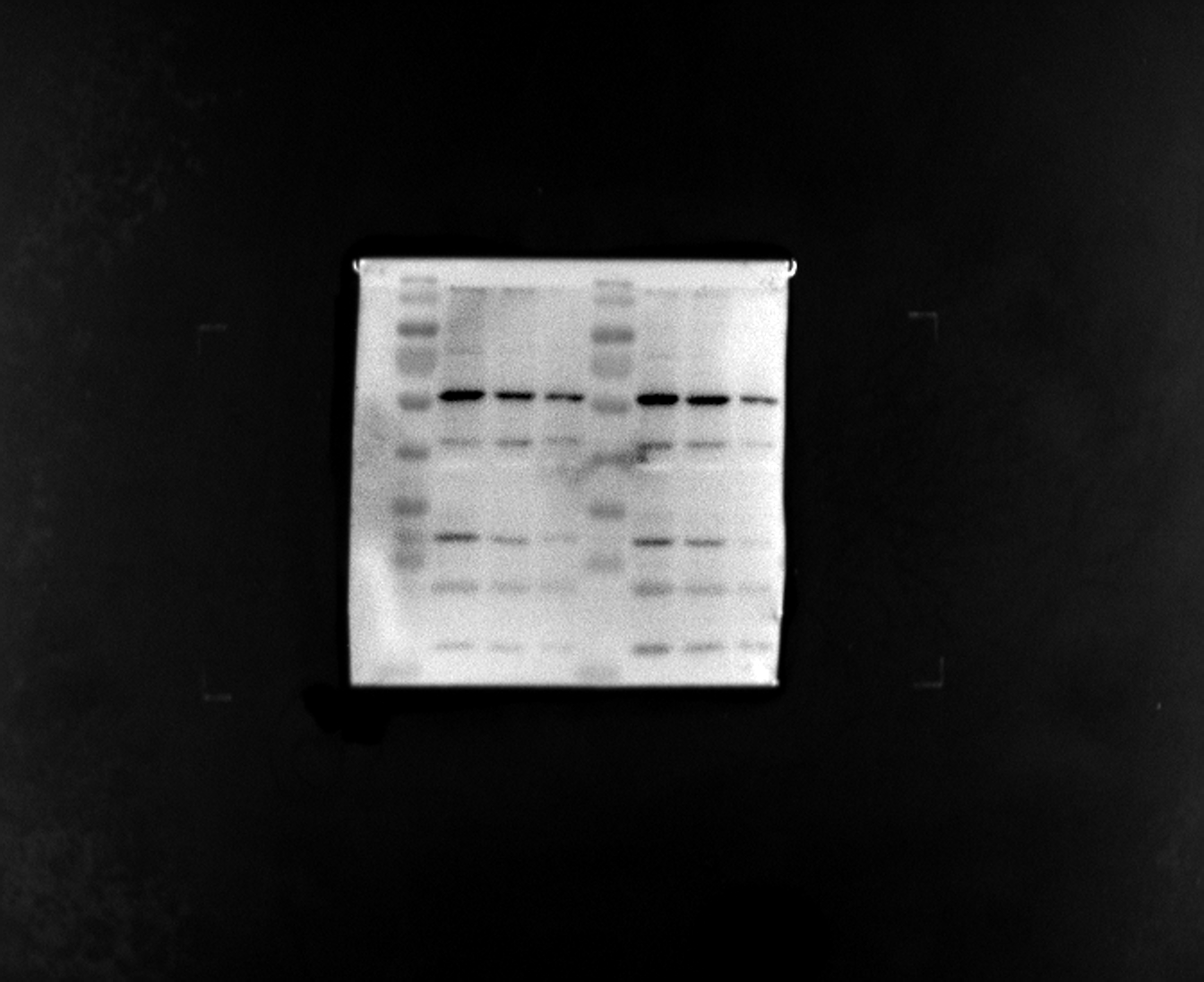

Supplement: Supplementary file 1 [file biology-11-00141-s001.zip › biology-1512795-supplementary/Supplementary File/biology-1512795 File S1/ETS/ETS-1.Tif]

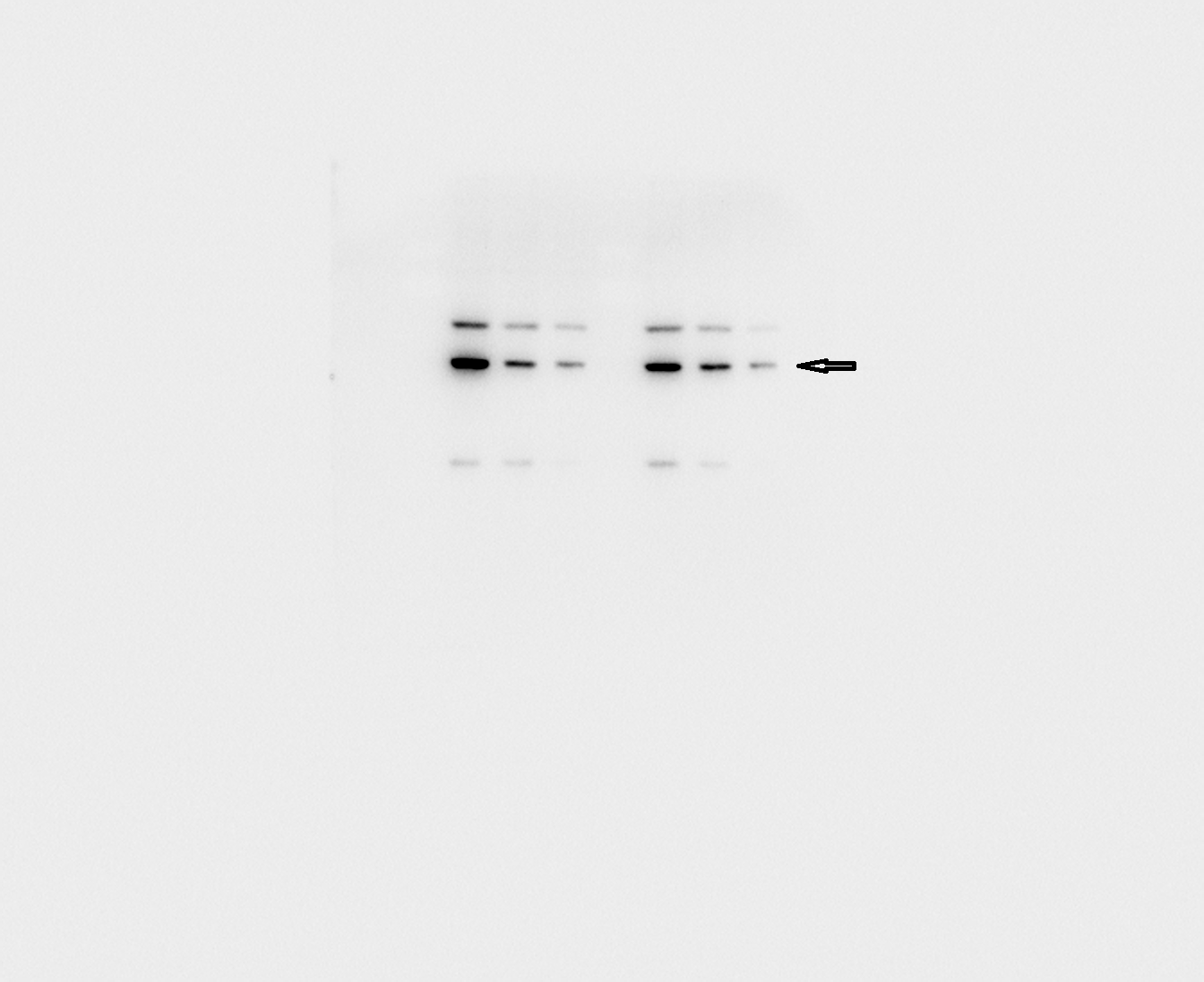

Supplement: Supplementary file 1 [file biology-11-00141-s001.zip › biology-1512795-supplementary/Supplementary File/biology-1512795 File S1/GABPA/3-GABPA-1.Tif]

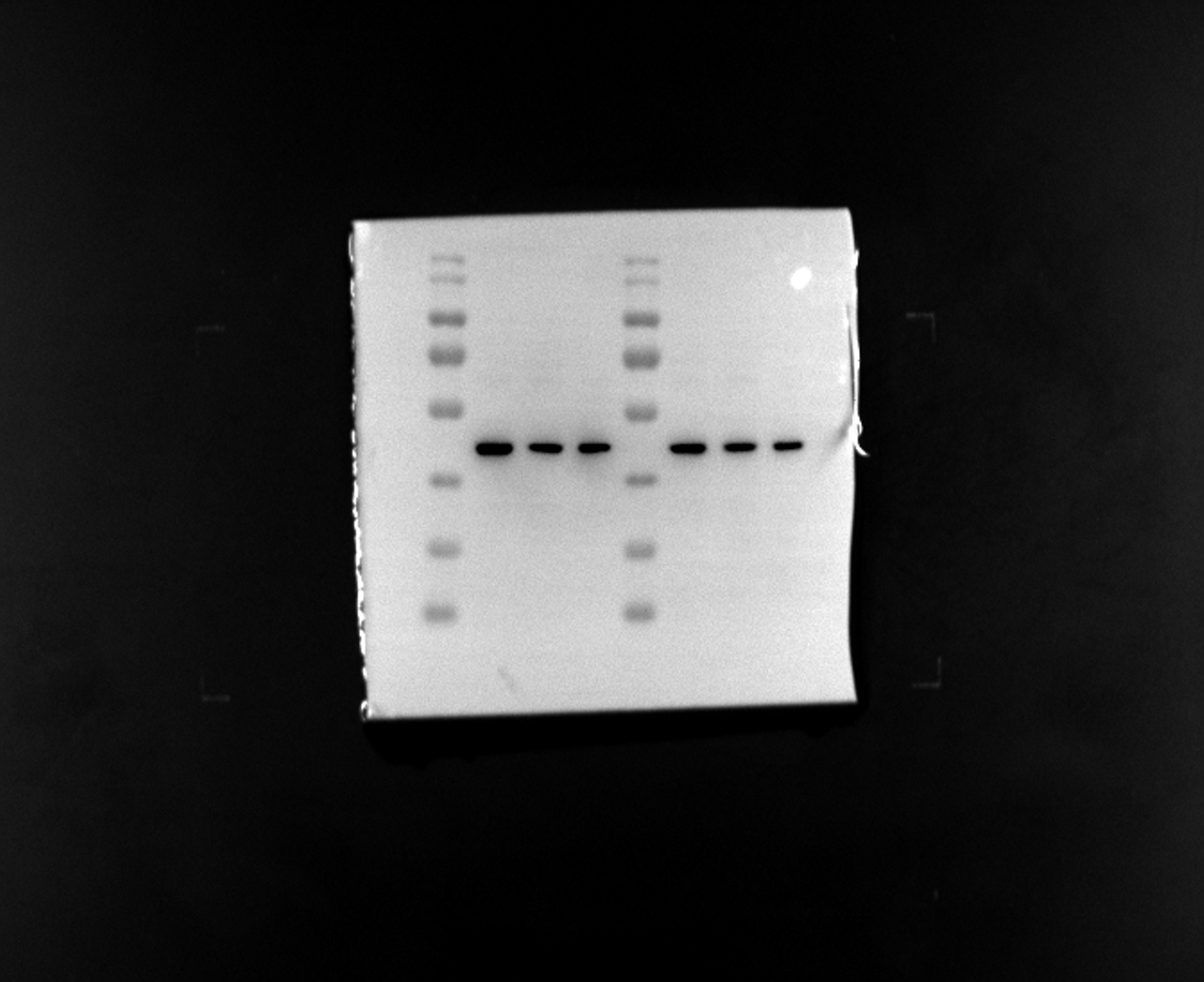

Supplement: Supplementary file 1 [file biology-11-00141-s001.zip › biology-1512795-supplementary/Supplementary File/biology-1512795 File S1/GABPA/GABPA-1 marker.Tif]

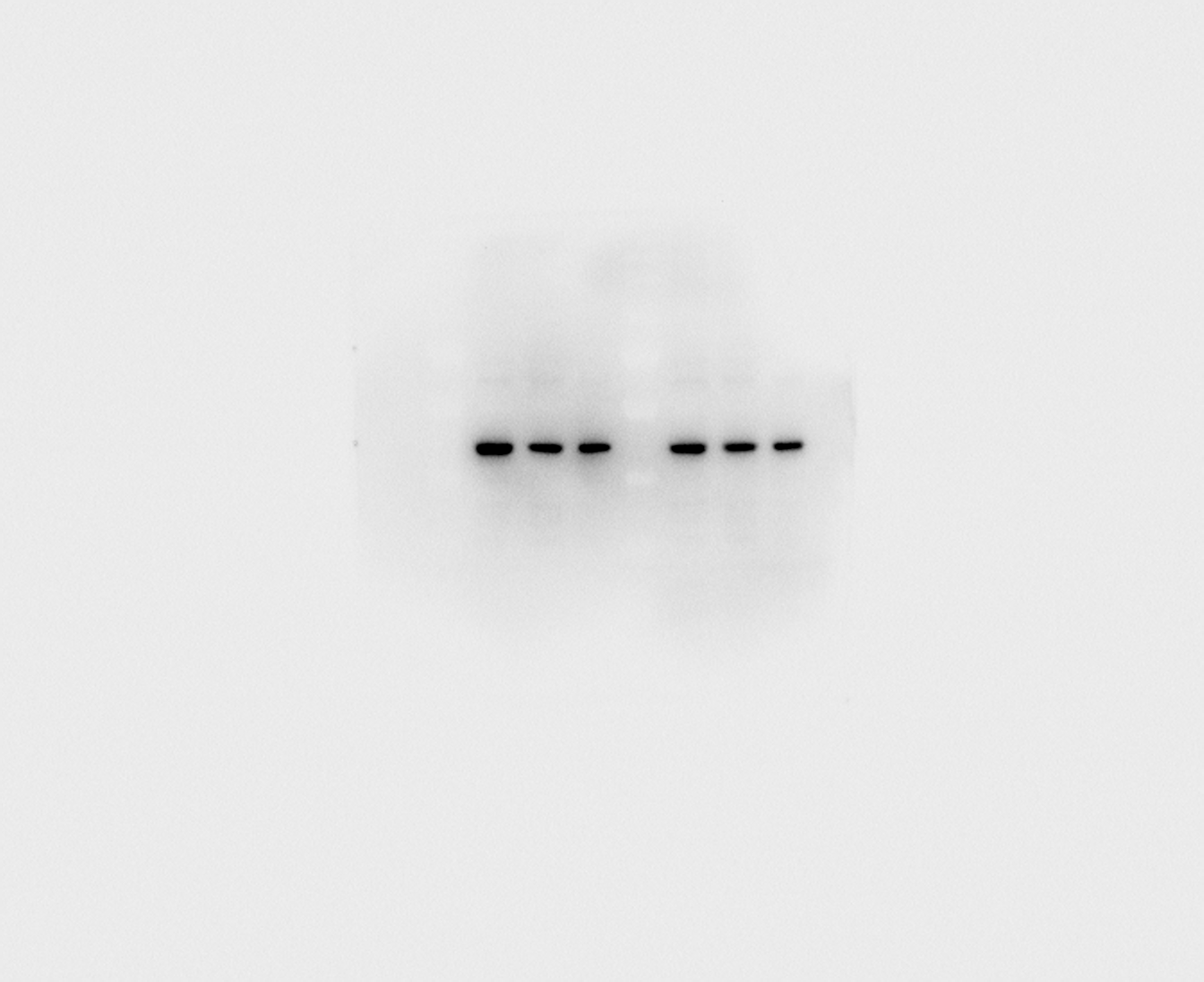

Supplement: Supplementary file 1 [file biology-11-00141-s001.zip › biology-1512795-supplementary/Supplementary File/biology-1512795 File S1/GABPA/GABPA-actin-1.Tif]

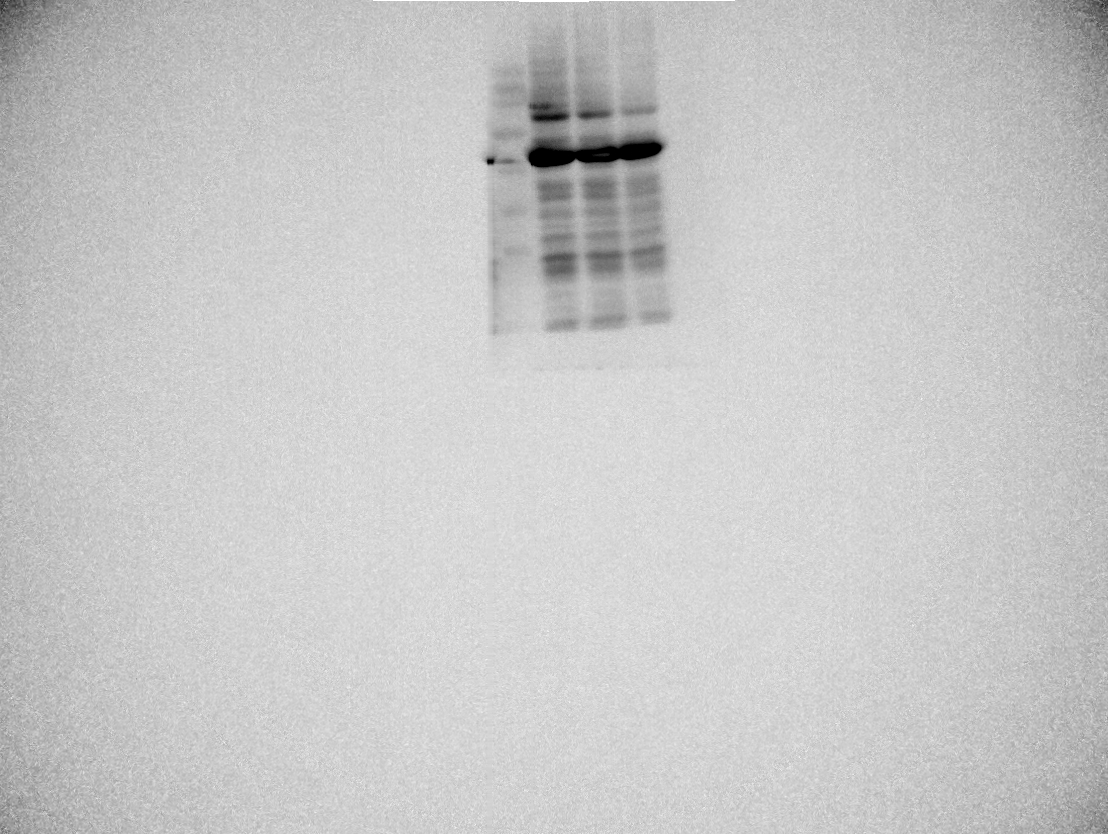

Supplement: Supplementary file 1 [file biology-11-00141-s001.zip › biology-1512795-supplementary/Supplementary File/biology-1512795 File S1/GABPB1/GABPB1 actin.jpg]

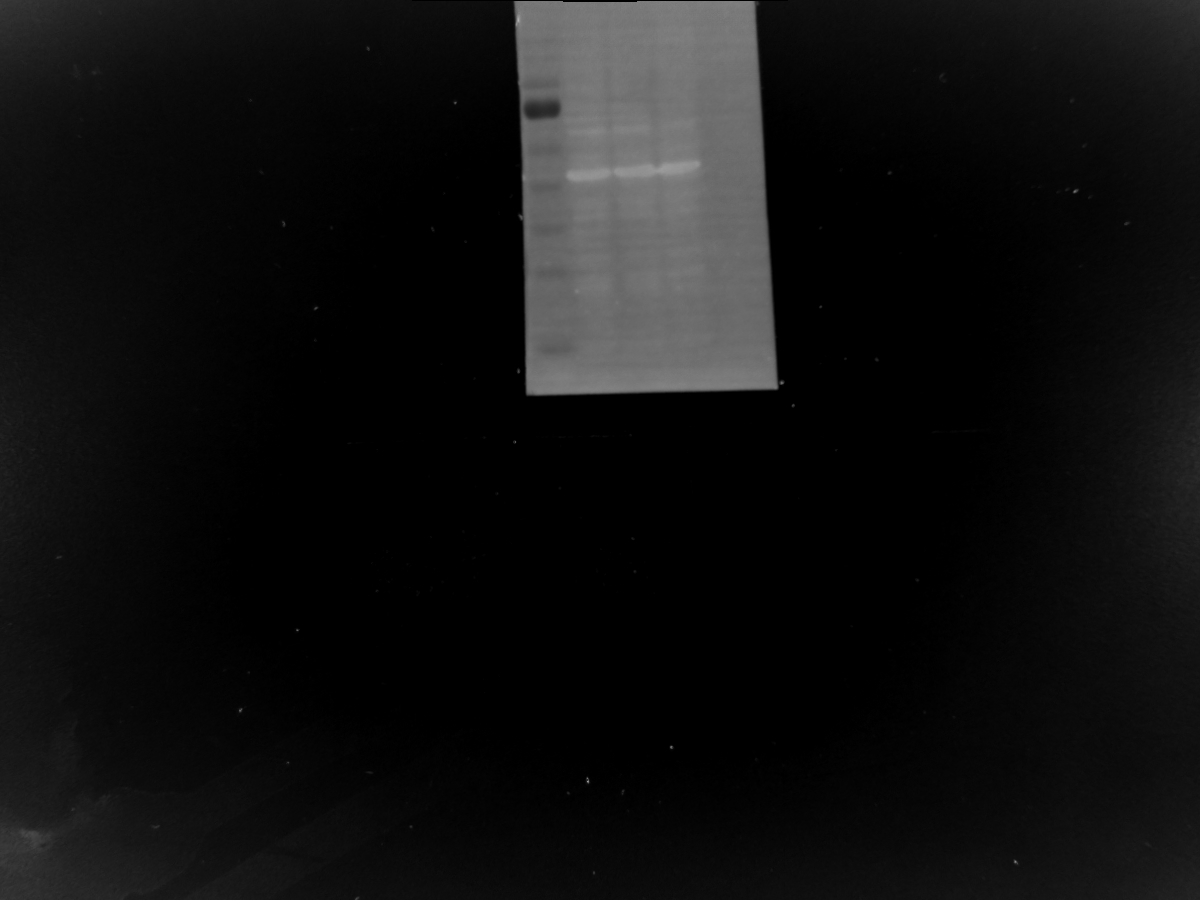

Supplement: Supplementary file 1 [file biology-11-00141-s001.zip › biology-1512795-supplementary/Supplementary File/biology-1512795 File S1/GABPB1/GABPB1 marker.jpg]

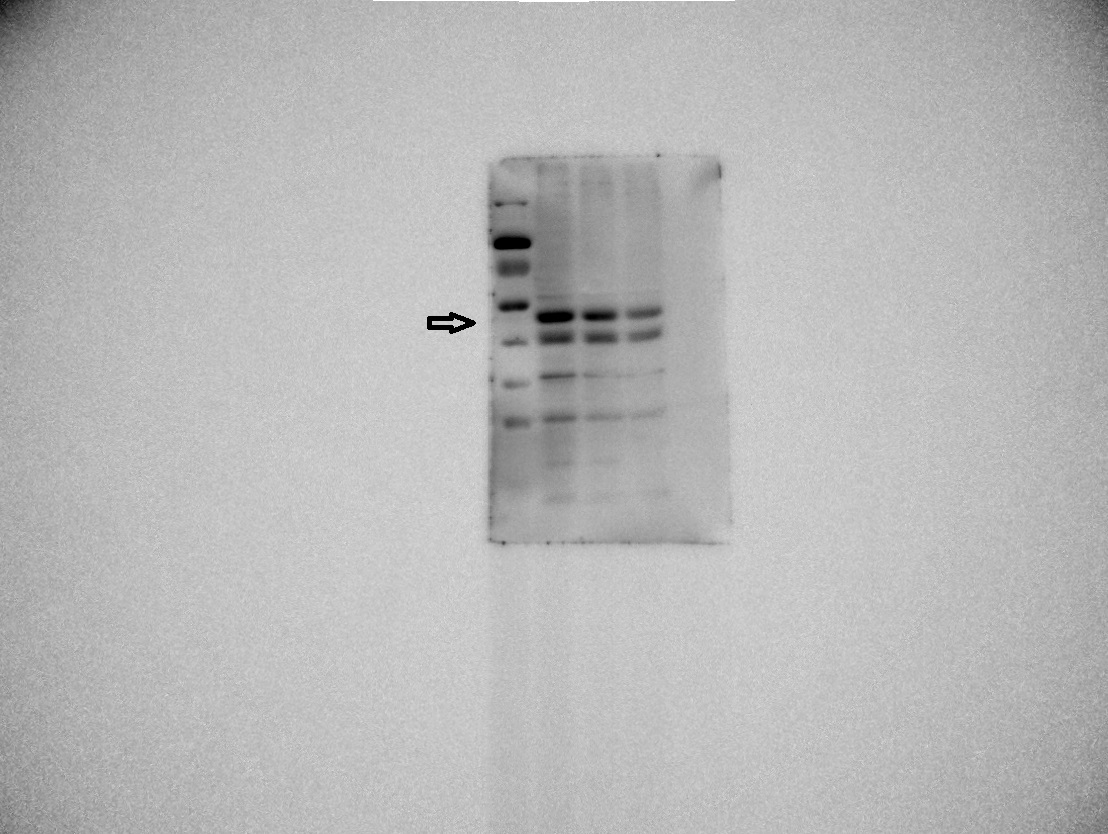

Supplement: Supplementary file 1 [file biology-11-00141-s001.zip › biology-1512795-supplementary/Supplementary File/biology-1512795 File S1/GABPB1/GABPB1.jpg]

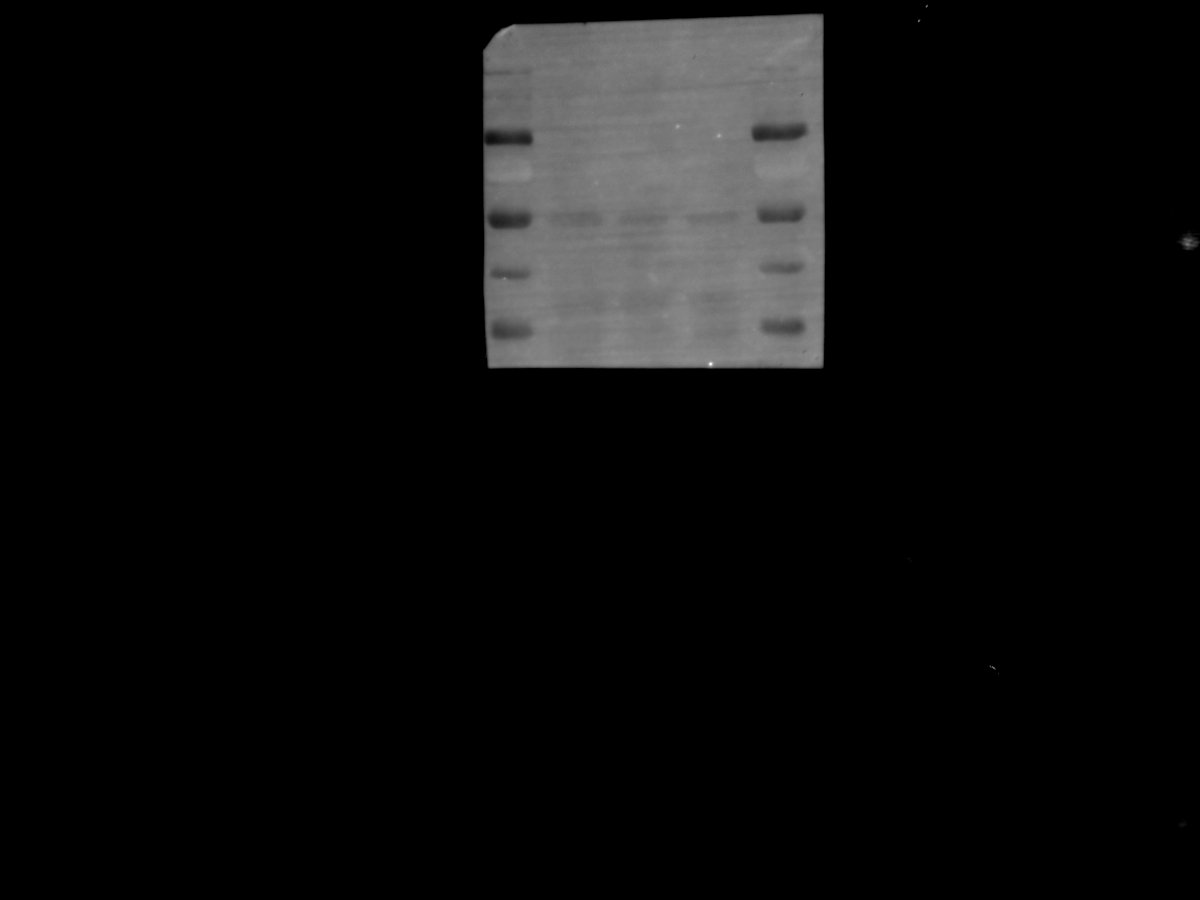

Supplement: Supplementary file 1 [file biology-11-00141-s001.zip › biology-1512795-supplementary/Supplementary File/biology-1512795 File S1/PTEN/PTEN-3 marker.jpg]

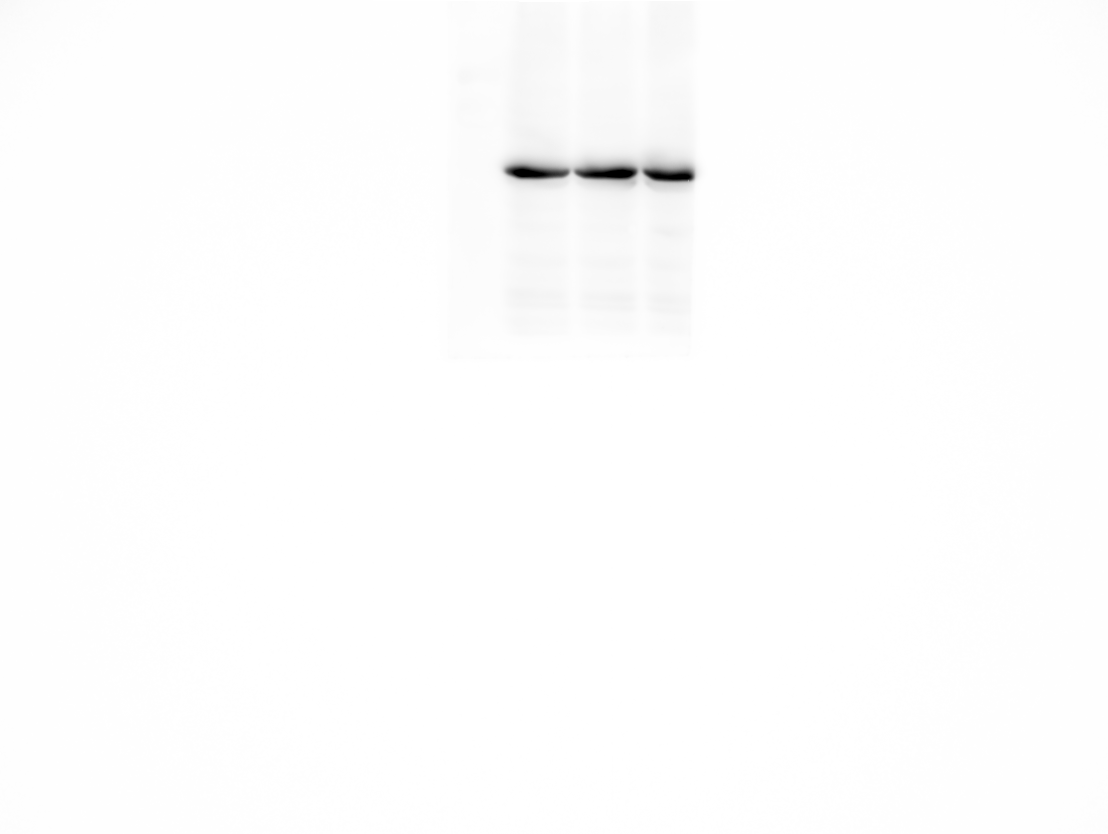

Supplement: Supplementary file 1 [file biology-11-00141-s001.zip › biology-1512795-supplementary/Supplementary File/biology-1512795 File S1/PTEN/PTEN-3 tubulin.tif]

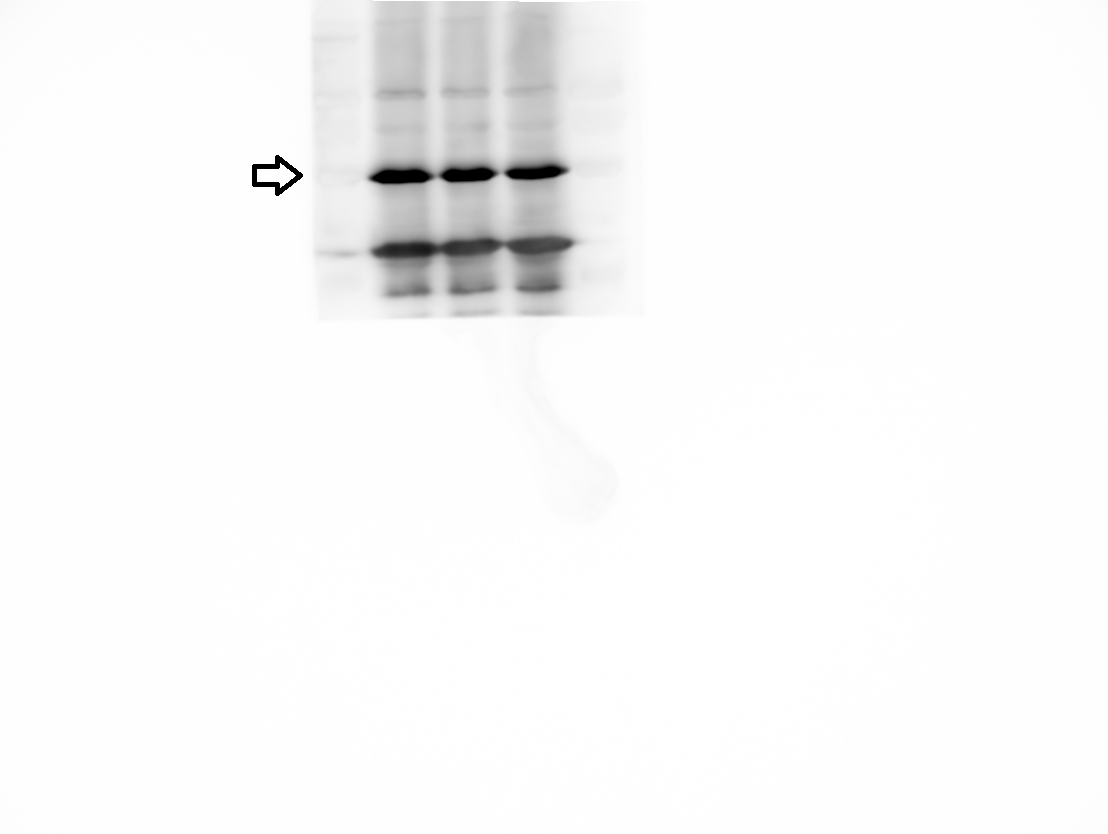

Supplement: Supplementary file 1 [file biology-11-00141-s001.zip › biology-1512795-supplementary/Supplementary File/biology-1512795 File S1/PTEN/PTEN-3.tif]

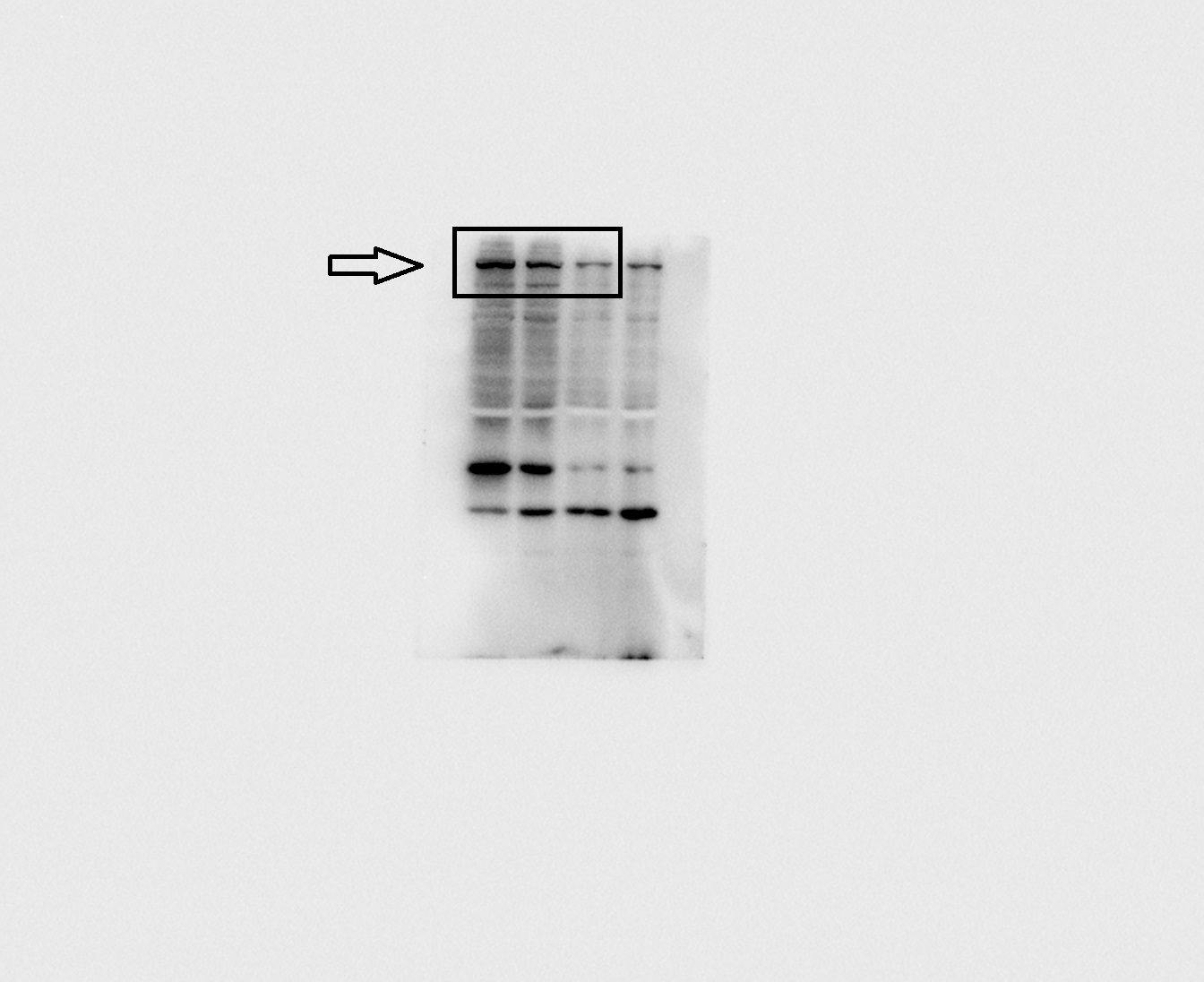

Supplement: Supplementary file 1 [file biology-11-00141-s001.zip › biology-1512795-supplementary/Supplementary File/biology-1512795 File S1/TERT/TERT-1.jpg]

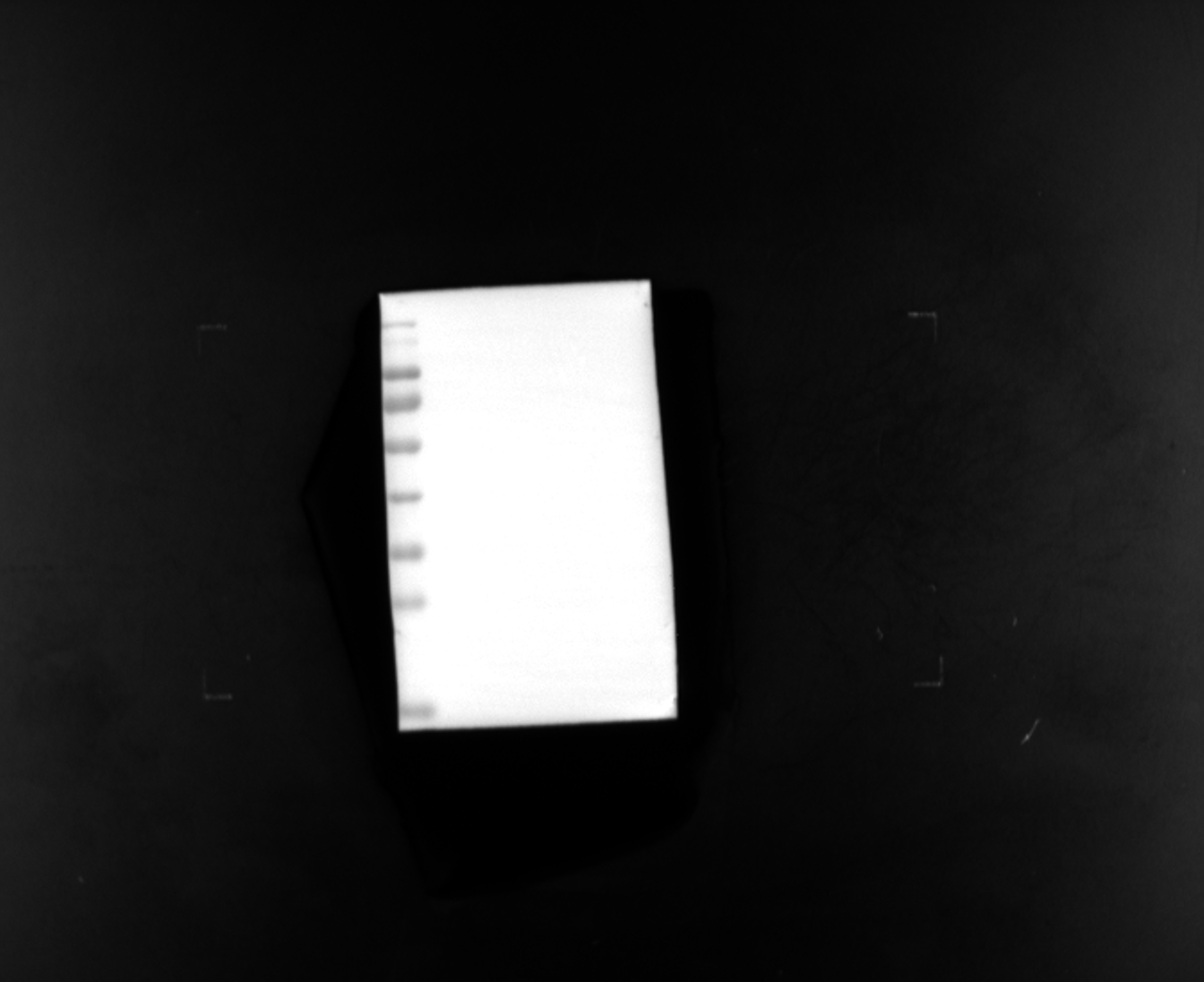

Supplement: Supplementary file 1 [file biology-11-00141-s001.zip › biology-1512795-supplementary/Supplementary File/biology-1512795 File S1/TERT/TERT-marker.jpg]

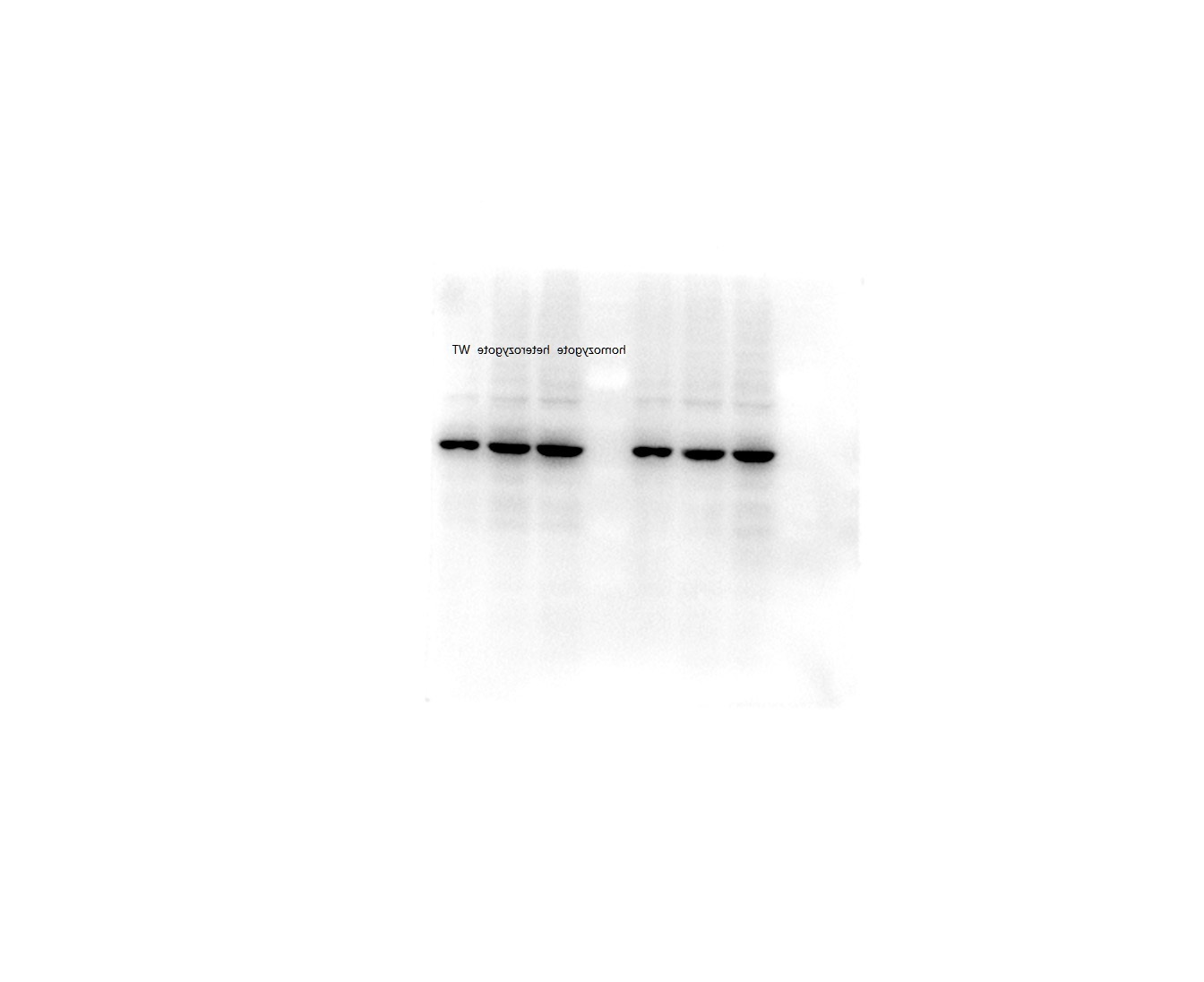

Supplement: Supplementary file 1 [file biology-11-00141-s001.zip › biology-1512795-supplementary/Supplementary File/biology-1512795 File S1/VDAC1/VDAC1 actin-1.jpg]

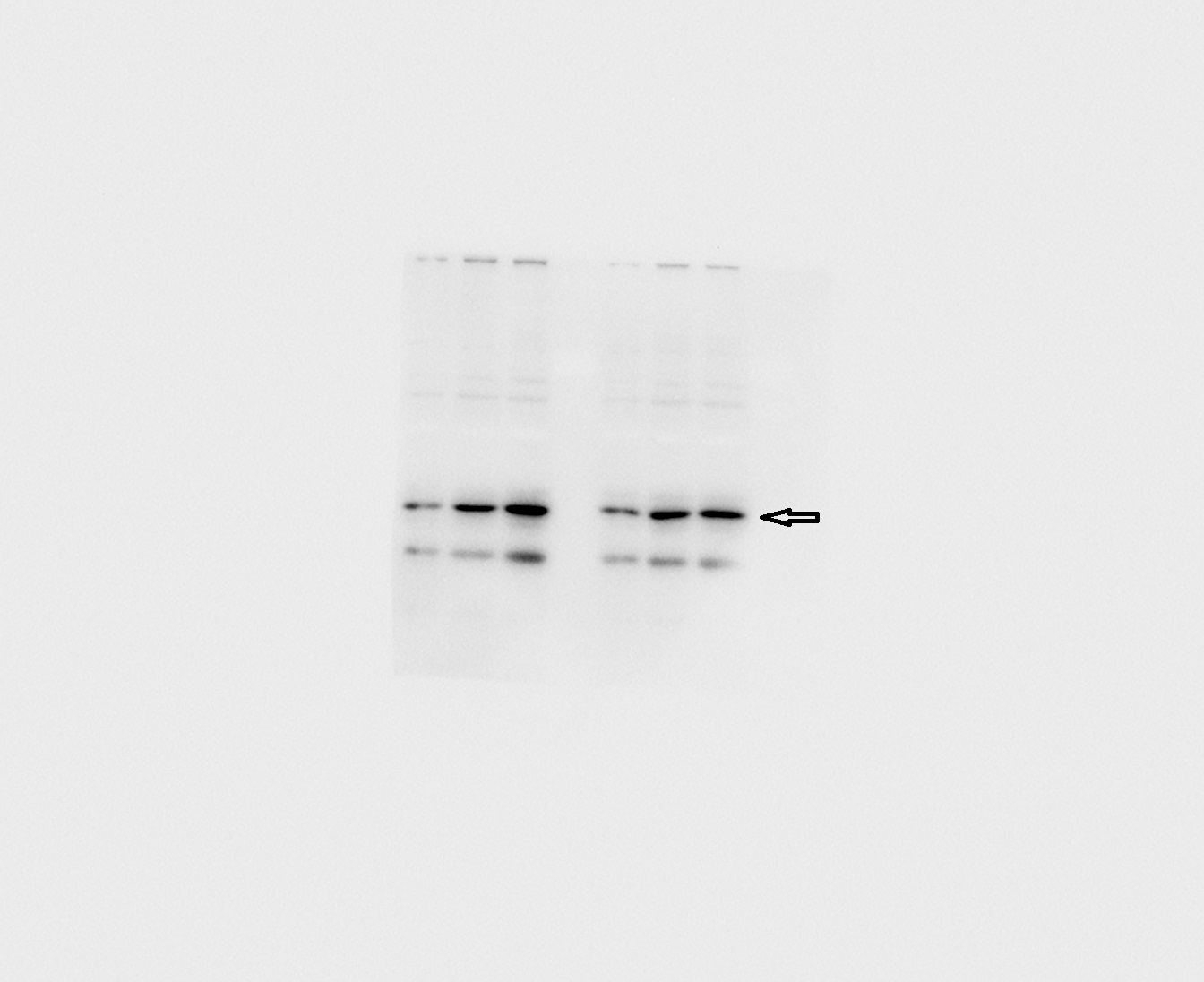

Supplement: Supplementary file 1 [file biology-11-00141-s001.zip › biology-1512795-supplementary/Supplementary File/biology-1512795 File S1/VDAC1/VDAC1-1.jpg]
